# Harnessing the Enzymatic Potential of Indigenous Yeast Strains: Screening and Evaluation for Biocontrol and Oenological Advancements

**DOI:** 10.3390/microorganisms14030705

**Published:** 2026-03-21

**Authors:** Rowland Adetayo Adesida, Jan Reščič, Lorena Butinar, Melita Sternad Lemut

**Affiliations:** Wine Research Centre, University of Nova Gorica, Vipavska 13, SI-5000 Nova Gorica, Slovenia; rowlyadesida@yahoo.co.nz (R.A.A.); jan.rescic@ung.si (J.R.)

**Keywords:** indigenous wine yeasts, *Saccharomyces* yeasts, non-*Saccharomyces* yeasts, hydrolytic enzyme activities, *β*-glycosidase activities, *β*-lyase activity, sulfite reductase activity, biocontrol yeasts, *Botrytis cinerea*, integrated disease management

## Abstract

The growing emphasis on sustainability, regional distinctiveness, and spontaneous fermentation in winemaking necessitates a more comprehensive understanding of local yeast populations and their functional mechanisms. In total, 115 indigenous yeast strains were examined for their enzymatic activities of potential vitivinicultural significance. The yeasts were screened for chitinase activity (biocontrol potential), glycosidase activity (terpene release), *β*-lyases (thiol release), and sulfite reductases (off-flavor formation), followed by quantitative analysis of the selected subsets. Yeasts were further evaluated for inhibition of fungal mycelial growth, VOC-mediated inhibition, and tolerance to commonly applied fungicides. Pre-field selection was refined using the niche overlap index and grapevine leaf disc assay. The results confirmed chitinolytic activity in four species; all strains exhibited hydrolase activities, with *H. uvarum* 116 displaying the highest cell-associated activity (6.32 U/g), while *T. delbrueckii* Sut94 showed the highest extracellular activity (1.36 U/g). *β*-glucosidase and *β*-lyase activities were widespread, whereas hydrogen sulfide production was infrequent. *P. guilliermondii* ZIM 624 showed the most comprehensive overall enzymatic profile, together with strong inhibition patterns. A field trial on Pinot cultivars (*V. vinifera* L.) further evaluated *P. guilliermondii* ZIM 624 within an integrated disease management approach, with responses being more pronounced in ‘Pinot noir’ than in ‘Pinot gris’.

## 1. Introduction

Yeasts are the principal microorganisms responsible for alcoholic fermentation (AF), a major process and signature step in winemaking. Vineyard-associated communities comprise diverse yeast species and strains that coexist and interact within grape must during spontaneous fermentation and under controlled inoculation with *Saccharomyces* and non-*Saccharomyces* starters. These interactions shape key metabolic processes that influence wine composition and quality [1,2,3,4]. As integral components of vineyard ecosystems, indigenous yeasts are shaped by the interplay of local and regional environmental conditions, soil characteristics, grapevine cultivars, surrounding biota, and human practices—factors that may ultimately contribute to the site-specific distinctiveness of regional wines [5].

The use of selected *Saccharomyces* strains is common in modern winemaking to achieve faster, more reliable initiation of fermentation and improved process control. In recent decades, the selection criteria for commercial starters have expanded beyond fermentation robustness to include aroma modulation, stress tolerance, and compatibility with specific grape varieties and winemaking styles [6,7]. In parallel, the interest in non-*Saccharomyces* yeasts has increased substantially. Once regarded primarily as undesirable or technologically problematic, these yeasts are now recognized for their broad metabolic diversity, enzymatic potential, and capacity to influence wine composition and sensory attributes [1,8,9,10,11]. Several species, including *Lachancea thermotolerans*, *Metschnikowia pulcherrima*, *Schizosaccharomyces pombe*, *Pichia kluyveri*, and *Torulaspora delbrueckii*, have been commercially employed or are under active investigation [12,13,14].

Beyond their oenological relevance, yeasts have attracted increasing attention in viticulture due to their antagonistic activity against grapevine pathogens [11,15]. Yeast-based biocontrol agents combine environmental compatibility, low toxicity, and adaptation to plant-associated habitats, positioning them as promising alternatives or complements to synthetic fungicides in sustainable vineyard management [16,17,18,19].

*Botrytis cinerea* Pers. is the causal agent of gray mould (*Botrytis* bunch rot), one of the most economically significant grapevine diseases worldwide [20,21]. The pathogen predominantly infects ripening berries, and disease severity is strongly influenced by humidity, temperature, and cluster compactness, making tight-clustered and early-ripening cultivars, such as Pinot varieties, particularly susceptible to infection [22,23]. In these cultivars, disease pressure coincides with distinctive phenolic and aromatic profiles that may be modulated by specific yeast strains during fermentation [24,25,26].

Numerous yeast species have been reported to inhibit grapevine pathogens, including *B. cinerea* through diverse mechanisms that define their biocontrol potential and may be further enhanced by consortium-based approaches combining complementary antagonistic modes of action [19,21,27,28,29,30,31,32]. Competition for space and nutrients represents a primary strategy, whereby efficient resource acquisition limits pathogen development, resulting in reduced fungal growth and niche overlap [33,34,35]. In addition, indirect antagonism mediated by volatile organic compounds (VOCs) enables pathogen suppression without physical contact, highlighting the role of yeast secondary metabolism, and several yeast species have been shown to inhibit *B. cinerea* through VOC release [18,35,36,37]. The effectiveness of these mechanisms ultimately depends on the yeast’s capacity to tolerate vineyard-relevant stresses, including copper exposure and commercial fungicide treatments [32,38,39].

Despite this growing body of research, the development of yeast-based biocontrol strategies remains constrained by fragmented evaluation across experimental systems and application contexts [21]. Many studies rely on simplified in vitro assays or focus on single antagonistic mechanisms, whereas fewer incorporate plant-based bioassays and vineyard-relevant stress factors that better approximate field conditions, hindering a comprehensive understanding of the interactions among the host, pathogen, and biocontrol agents [40,41].

Consequently, translating promising laboratory findings into consistent vineyard performance remains a significant bottleneck. Moreover, vineyard biocontrol and vinification are typically investigated separately, and studies that jointly assess biocontrol performance under vineyard conditions alongside fermentation-related functionality within the same yeast collection remain limited [5,11]. Such compartmentalization overlooks the potential of vineyard-associated yeasts to fulfill dual roles across both agronomic and oenological frameworks.

The enzymatic repertoire of wine-related yeasts represents an important functional link between these two domains.

Enzymes such as chitinases may contribute to the degradation of fungal cell walls and pathogen suppression; glycosidases and *β*-glucosidases are associated with the release of aroma compounds from grape precursors; *β*-lyases participate in volatile thiol release; and sulfite reductases are linked to off-flavor formation [42,43,44,45,46].

Although enzymatic profiling alone does not confirm functional performance in either biocontrol or vinification, it provides valuable indicators of yeast functional potential and supports the selection of candidate strains for further evaluation under vineyard- and fermentation-relevant conditions.

In this context, we aimed to comprehensively evaluate indigenous *Saccharomyces* and non-*Saccharomyces* yeast strains isolated from Slovenian vineyards and wines for their potential relevance in both vineyard disease management and vinification. Using an integrated framework spanning laboratory assays, plant-based bioassays, and field evaluation, we examined their interactions with *B. cinerea* together with vinification-related functional traits. By considering these dimensions within the same yeast collection, the study seeks to address the gap between simplified laboratory screening and vineyard-oriented evaluation and to identify candidates for future sustainable strategies linking disease management and winemaking.

## 2. Materials and Methods

### 2.1. Yeast Strains and Phytopathogenic Fungal Strain

This study employed 115 indigenous yeast strains from 30 species across 17 genera sourced from two separate Slovenian microbial repositories: (1) the Yeast Bank of Wine Research Centre, University of Nova Gorica (YBWRC), and (2) the Repository of Industrial Microorganisms (ZIM), Ljubljana. YBWRC yeast isolates derived from a previous research exploring yeast biodiversity on grape berries [38,47], whereas ZIM provided yeast strains previously characterized as putative biocontrol agents [48]. The phytopathogenic fungus *B. cinerea* F61 was obtained from the ZIM. Further details regarding the origin of the selected yeast and fungal isolates are presented in Table S1 (Supplementary Materials).

### 2.2. Cell Wall–Degrading Enzyme Assays

Yeast strains were first prescreened qualitatively for chitinase production, and selected strains were subsequently used for quantitative determination of endo- and exochitinase and *β*-1,3-glucanase activities.

#### 2.2.1. Chitinase Assays

*Qualitative screening on BMC agar*. Chitinase activity was screened on the BMC (Base Medium with Chitin) agar described by Agrawal and Kotasthane (2012), in which chitin is the sole carbon source and bromocresol purple serves as pH indicator [49]. The medium composition consisted of agar (15.0 g/L), colloidal chitin (4.5 g/L), bromocresol purple (0.15 g/L), MgSO_4_ (0.15 g/L), KH_2_PO_4_ (2.0 g/L), (NH_4_)_2_SO_4_ (3.0 g/L), citric acid monohydrate (1.0 g/L), and Tween 80 (200 μL), adjusted to pH 4.7 [49,50].

The yeast preculture for assay inoculation was prepared by transferring a 3-day-old colony, grown on Yeast Extract–Peptone–Dextrose (YPD; 20 g/L agar, 10 g/L yeast extract, 20 g/L glucose and 20 g/L peptone) plates, into a liquid YPD medium and incubated for a day at 25 °C and agitated at 250 rpm. Thereafter, yeast biomass was harvested by centrifugation (10 min, 680 g, RT), resuspended in 0.85% NaCl and subjected to another centrifugation and the yeast concentration was adjusted to OD_640_ = 1 (optical density at 640 nm absorbance).

A suspension aliquot (5 μL, OD_640_ = 1) of each tested strain was spot-inoculated onto BMC plates. The inoculated plates underwent incubation at 25 °C for a duration of seven days. Subsequently, the presence of a purple-colored zone was evaluated. Yeast isolates demonstrating this visual change were designated as chitinase-positive [49,50]. The experiments were performed in triplicate.

*Endo- and exo-chitinase activity assays.* For quantitative assays, colloidal chitin was prepared from shrimp shell chitin following previously described protocols [49,51], and *B. cinerea* cell-wall preparations (CWP) were obtained from 5-day-old cultures grown in malt extract broth according to a previously reported method [52].

Endo- and exo-chitinase activities were quantified in two selected yeast strains, *W. anomalus* S138 and *P. guilliermondii* ZIM 624. Precultures were prepared in liquid YPD as described above, harvested, washed, and used to inoculate 100 mL Erlenmeyer flasks containing 30 mL Lilly–Barnett minimal salt medium [53] supplemented with 2 mg/mL CWP and either glucose or sucrose as the sole carbon source. Flasks were incubated at 25 °C on a rotary shaker (150 rpm), and samples were collected after 0, 24, 48, 72, 96, and 120 h. Cultures were centrifuged (7000× *g*, 8 min, 4 °C), and the supernatants were used as crude enzyme preparations.

Exo-chitinase activity was determined according to a previously established protocol [54]. Thus, a reaction mixture was prepared by combining 500 µL of enzyme supernatant solution and 500 µL of colloidal chitin (5 mg/mL), containing sodium azide (1.2 µmol/L) and sodium acetate (56 µmol/L).

The assay for endo-chitinase activities followed the established methodology delineated in a previous study [55]. In brief, 500 µL of colloidal chitin (5 mg/mL), 100 µL of 3% (*w*/*v*) cytohelicase from previously desalted snail guts (Sigma-Aldrich, St. Louis, MO, USA), and 100 µL of KH_2_PO_4_ buffer (1 mol/L, pH 7.1) were combined with 500 µL of enzyme supernatant, and the enzyme-substrate mixture was kept at 37 °C for 2 h with constant stirring. The supernatant was collected from the mixture after 8 min of centrifugation at 7000× *g*. The content of reducing sugars was assessed using a D-glucosamine assay kit (Megazyme K-GAMINE 04/18; Megazyme International Ltd., Bray, Ireland), while the protein concentration in the enzyme solution was measured with the Invitrogen QubitTM Protein Assay Kit (Thermo Fisher Scientific, Invitrogen, Eugene, OR, USA) [55]. The specific activities (SA) were presented as micromoles of N-acetyl-D-glucosamine per milligram of protein per hour [56]. Four replicates were utilized for each experimental setup.

#### 2.2.2. β-1,3-Glucanase Assay

*β*-1,3-glucanase activity was evaluated in *W. anomalus* S138 and *P. guilliermondii* ZIM 624, previously selected based on their chitinase performance. The two strains were inoculated into modified Lilly–Barnett medium [53] prepared, incubated, and processed as described above for the chitinase assay (Section 2.2). After incubation, cultures were centrifuged, and the resulting supernatants were used for *β*-1,3-glucanase determination.

A reaction mixture was formulated by combining equal volumes (250 µL) of the culture filtrate and CH_3_COOK buffer (50 mM, pH 5.0) containing laminarin (2.5 mg/mL). This mixture then underwent incubation for 2 h at 40 °C. Subsequently, the activities of *β*-1,3-glucanase were determined by measuring the free glucose released from laminarin with the help of glucose oxidase kit (Megazyme K-GLUC 02/18; Megazyme International Ltd., Bray, Ireland). The enzyme solution’s protein concentration was measured as previously outlined for the endo-chitinase activity assay (Section 2.2.1). SA was expressed as micromoles (µmol) of glucose per milligram (mg) of protein per h [57,58]. Each experimental setup was conducted with four replicates.

### 2.3. Glucoside Hydrolase Assays

Glucoside hydrolase activities related to aroma release were assessed by qualitative screening on five *β*-D-glucosidic substrates and by quantitative determination of *β*-glucosidase activity using p-nitrophenyl-*β*-D-glucopyranoside (pNPG) as substrate.

*Qualitative screening on arbutin (A), esculin (E), cellobiose (C), salicin (S), and 4-methylumbelliferyl-β-D-glucoside (4-MUG)*. Yeast strains were recovered from cryo-stocks (15% glycerol) by growth in 200 μL YPD at 25 °C for 24 h with shaking. Cells were harvested by centrifugation (900× *g*, 10 min), washed once with 0.85% (*w*/*v*) NaCl, and 2 μL of the resulting suspensions were spotted onto square plates (10 cm × 10 cm) containing the Yeast Nitrogen Base (YBN) (0.67% *w*/*v*) (Biolife, Milano, Italy) supplemented with one of five *β*-D-glucosidic substrates: (1) arbutin (0.5% *w*/*v*); (2) esculin (0.05% *w*/*v*); (3) cellobiose (0.5% *w*/*v*); (4) salicin (0.5% *w*/*v*), and (5) 4-MUG (0.04% *w*/*v*) [59]. The pH of each medium was adjusted to 5. Following incubation at 25 °C, the plates were assessed for growth after a period of 5 days. The presence of positive hydrolase activities on 4-MUG plates was ascertained through the observation of a fluorescent halo surrounding yeast colonies after a 5-day incubation period. This halo, visible under UV-radiation, signified the liberation of 4-methylumbelliferone [59]. Each experimental setup was conducted with four replicates.

*Quantitative β-glucosidase activity on pNPG.* Strains that showed positive or strong hydrolase activity on at least one of the substrates in the qualitative assay were selected for quantitative determination of extracellular and cell-associated *β*-glucosidase activity, using pNPG as substrate [60]. Precultures were prepared by inoculating a small amount of pre-grown cells into 10 mL YPD liquid (pH 5.0) and incubating at 25 °C for 24–72 h, depending on strain growth rate. These precultures were used to inoculate fresh YPD to 1/5 of the flask volume and a final cell density of approximately 1 × 10^6^ cells/mL, followed by incubation at 20 °C, 150 rpm, until late exponential phase.

To prepare the samples for the extracellular activity assay, a 1 mL aliquot of yeast cell suspension was subjected to centrifugation conducted at 3000× *g* for a duration of 10 min at 4 °C and the resulting 200 µL supernatant was used to determine extracellular activities on pNPG [60].

For the purpose of cell-associated activity analysis, the yeast pellet underwent a washing procedure utilizing a cold solution of NaCl (7 g/L), followed by centrifugation at 3000× *g* for 10 min at 4 °C. After discarding the supernatant, 200 µL of McIlvaine buffer, containing citric acid (0.1 mol/L) and Na_2_HPO_4_ (0.2 mol/L); pH 5) was incorporated to the pellet [60].

The quantitative determination of extracellular and cell-associated activities involved combining 200 µL pNPG solution (5 mmol/L pNPG in McIlvaine buffer, pH 5) with either 200 µL supernatant or the pellet suspended in 200 µL of buffer. The mixture was incubated for 1 h at 30 °C. Following incubation, 800 µL carbonate buffer (0.2 mol/L, pH 10.2) was added, and the samples were centrifuged at 4 °C and 3000× *g* for 10 min. The release of p-nitrophenol (pNP), resulting in an increase in absorbance, was monitored spectrophotometrically at 400 nm. Control samples were prepared for the substrate and various fractions, as previously described by Daenen et al. (2008) [60]. Dry weight evaluation involved filtering 20 mL of yeast strain cell suspension through a previously weighted 0.45 µm filter paper and drying at 100 °C until a constant weight was achieved. Each experimental setup was conducted with four replicates.

### 2.4. β-Lyase Activity Assays

*Qualitative screening on YCB–SMC medium.* Qualitative *β*-lyase activity was assessed following the established protocol [46]. The Yeast Carbon Base (YCB) medium employed in this investigation comprised 1.2% YCB (*w*/*v*) (Biolife, Milano, Italy) supplemented with 0.01% pyridoxal-5-phosphate (*w*/*v*), 0.1% S-methyl-L-cysteine, and 2% agar (*w*/*v*). The supplemented medium was designated as YCB-SMC. The medium’s pH was set to 3.5. To prevent agar hydrolysis, the preparation involved blending previously autoclaved agar solution with the solution of the remaining ingredients, which were previously filter sterilised [46,50]. Before blending, the pH was adjusted to 3.5 by adding HCl. The resultant YCB-SMC medium was dispensed into square (10 cm × 10 cm) plates. To eliminate false-positive outcomes attributable to cellular reserves, yeast cultures were transferred from 3-day-old YCB-SMC medium to fresh YCB-SMC medium, and the presence of *β*-lyase activity was confirmed by substantial colony growth observed on the square plates following a 2–3 day incubation period at 28 °C [46,50]. All experiments were performed in triplicate.

*Cysteine β-lyase activity assay*. The experimental protocol was based on a previously reported methodology [61], with minor modifications in the cell-disruption step [50]. In summary, after overnight cultivation in YPD medium, the yeast cell suspension (8 mL) underwent centrifugation for 3 min at 4000× *g*, followed by two washes with 1 mL Milli-Q water. The yeast cells were subsequently resuspended in 1000 μL of the breaking buffer with pH 6.8, composed of 2% *v*/*v* Triton X-100, 100 mM Tris-Cl. Approximately 900 μL of glass beads (Sigma-Aldrich, St. Louis, MO, USA), which had been pre-treated with acid, were incorporated, and the mixture was subjected to vigorous disruption utilizing a mixer mill MM400 (Retsch, Haan, Germany) for 5 min at 4 °C while maintained on ice. The resulting suspension underwent centrifugation for 30 min at 16,000× *g* and 4 °C to obtain the supernatant, which was later used for enzyme assays [50,61].

The reaction mixture comprised cysteine (150 μL, 10 mM), pyridoxal 5′-phosphate (30 μL, 1 mM), and assay buffer (1245 μL, 20 mM phosphate buffer, 1 mM EDTA, pH 7.0). Aliquots of 75 μL yeast cell lysates were introduced, and the enzymatic reaction was allowed to proceed for a duration of 60 min at a temperature of 30 °C. After this period (1 h), 5 μL L-lactic dehydrogenase enzyme (2 U/μL Megazyme E-LLDHP, EC 1.1.1.27) (Megazyme International Ltd., Bray, Ireland) was introduced to the reaction mixture, promptly followed by the addition of 100 μL NADH (3 mM). For all observed reactions, the absorbance at 340 nm was quickly measured to reflect the relative levels of NADH. Subsequent to the A340 measurement at 60 min, the reactions (now containing L-lactic dehydrogenase and NADH) were allowed to continue, with A340 measurements taken at 75, 90, and 120 min to assess NADH consumption [61]. The assay was conducted in triplicate.

### 2.5. Qualitative Screening for Sulfite Reductase Activity

To screen for sulfite reductase activities, a microplate-based adaptation of the lead acetate method was utilized to assess H_2_S production, as outlined in previous research [62,63]. In brief, the yeast strains were cultivated in 96-well microplates, each well containing 1.17% YCB (*w*/*v*) medium (200 μL), supplemented with 4% glucose (*w*/*v*) and 0.5% ammonium sulfate (*w*/*v*). The cultures were then incubated for a period of 3 days at 28 °C, with continuous agitation at 200 rpm. The presence of H_2_S in the culture headspace was determined by examining the extent of discoloration (blackening) on lead acetate strips positioned above the microplate wells. These strips were treated beforehand by immersing Whatman filter paper in a lead acetate solution (0.1 M), followed by desiccation at 65 °C for 10 min [62,63]. All experiments were conducted in triplicate.

### 2.6. In Vitro Yeast-Mediated Fungal Mycelial Radial Growth Inhibition

The study on yeast-mediated inhibition of *B. cinerea* growth was conducted using the protocols outlined by Spadaro et al. (2010) [64] with minor adjustments. A loop of yeast culture, which had been grown for three days at 25 °C on WL nutrient medium (Biolife, Milano, Italy), was applied in a streak approximately 2 cm from the edge of a 9 cm diameter Petri dish, containing either YPDA or grape juice medium (GJM) [65]. A 50 µL aliquot of *B. cinerea* (10^4^ conidia/mL) suspension was applied centrally on the plate, positioned 3.2 cm from both the yeast outline and the edge of the agar plate [64]. The plates were incubated at 25 °C. During the initial week, growth was observed daily and reassessed after 30 days of incubation. When the mycelium reached the edge of the Petri dish, the following measurements were taken: (a) the inhibition distance, defined as the space between the yeast line and the advancing mycelial front of the filamentous fungus, and (b) the diameter of the filamentous fungal mycelium in the direction of the yeast line. Inhibitory activity was expressed as the percentage of inhibition distance relative to the combined distance of inhibition and pathogen growth. The study was performed in triplicate.

### 2.7. Inhibitory Activity of Yeast-Emitted Antifungal Volatile Organic Compounds (VOCs)

The antifungal activity of yeast-modulated volatile organic compounds (VOCs) against pathogen conidial germination was evaluated using a sealed double Petri dish assay (double-dish system), based on the protocols previously described by Di Francesco et al. (2015) and Ruiz-Moyano et al. (2020) with modifications to the medium used [66,67]. Plates containing YPDA and GJM were inoculated by evenly spreading 100 μL of a yeast suspension adjusted to an optical density of OD_640_ = 1. The plates were incubated at 25 °C for two additional days. After the incubation period, the lids of the plates were substituted with a base plate of malt extract agar (MEB supplemented with 20 g/L agar), which had been pre-inoculated at the central point with a conidial suspension. The assemblies were sealed with Parafilm^TM^. Throughout the assay, yeast and the phytopathogenic fungus (*B. cinerea*) were physically separated, allowing interaction exclusively via volatile compounds. The control consisted of plates inoculated solely with the pathogen. The results were quantified as the percentage of fungal growth inhibition. All experiments were performed in triplicate.

### 2.8. Yeast Tolerance to Copper and Other Commercial Fungicides

The tolerance of experimental yeast strains to conditions resembling those in vineyards, shaped by current disease control strategies, was tested on YPD agar medium (pH adjusted to 3.8) supplemented either with CuSO_4_ at concentrations of 2.5, 5.0, 7.5, 10.0, or 12.5 mM, or with commercial fungicides: (1) Switch^®^ 62.5 WG (0.1%) containing the active compounds cyprodinil and fludioxonil (Syngenta, Basel, Switzerland); (2) Rovral^TM^ Aquaflo (0.001%) containing the active compound iprodione (BASF, Basel, Switzerland); and (3) Banjo^®^ (0.01%) containing the active compound fluazinam (ADAMA Agriculture B.V., Leusden, The Netherlands). A 2 μL suspension of each yeast strain was spotted onto the plates, which were incubated at 25 °C for 7 days. All the experimental conditions were tested in triplicate.

### 2.9. Niche Overlap Index (NOI)

Niche Overlap Indexes were calculated following Nally et al. (2015) and La Penna et al. (2004) [34,68], with minor modifications. The growth media were prepared from YNB, adjusted to pH 5.5, and supplemented with a single carbon source at a concentration of 10 mM. For solid media, 20 g/L of agar was added. The 14 carbon sources selected for evaluation, which are representative of compounds commonly found in grapes, included alanine, arginine, asparagine, fructose, glucose, glutamic acid, glycine, lysine, malic acid, methionine, proline, raffinose, tartaric acid, and tyrosine. Plates were inoculated by applying 20 μL of either a fungal spore suspension (10^4^ spores/mL) or a yeast cell suspension (10^4^ cells/mL) at a single central inoculation point. The incubation was conducted in darkness at 25 °C for two weeks. Following incubation, the NOIs were calculated as the ratio between the number of carbon sources utilized by both yeast and the fungus and the total number of carbon sources utilized by the fungus. According to Wilson and Lindow (1994), NOI values greater than 0.90 suggest that organisms occupy the same ecological niche, indicating competitive exclusion [69]. Conversely, values below 0.90 imply that organisms inhabit distinct niches, allowing coexistence. All assays were conducted in triplicate.

### 2.10. Grapevine Leaf Disc Infection Bioassay

For yeast inocula preparation, yeast cultures were grown in liquid YPD at 25 °C and 150 rpm for 24 h, harvested by centrifugation (4000× *g*, 10 min, RT), washed with 0.85% NaCl, and resuspended in 16.7 mM KH_2_PO_4_ containing 25 mM glucose to a final concentration of 3 × 10^5^ cells/mL. While for *B. cinerea*, conidia were collected from PDA slants using 0.05% (*v*/*v*) Tween 80, filtered through sterile steel wool, centrifuged (10,000× *g*, 10 min, RT), and resuspended in 0.85% NaCl. The final conidial suspension (3 × 10^5^ conidia/mL) was prepared in 16.7 mM KH_2_PO_4_ with 25 mM glucose and pre-germinated at 22 °C for 2 h.

To set up the leaf disc assay, leaves from the grapevine variety ‘Pinot noir’ (*Vitis vinifera* L.) were collected, rinsed with a 0.85% NaCl solution, and then dried with blotting. Discs measuring 1.5 cm in diameter were cut from the leaves and positioned with the adaxial side facing up in 12-well plates, each containing 1.5 mL distilled water. Two 5 μL droplets were applied to each disc, which was then incubated at 22 °C in darkness. The experimental treatments comprised the pathogen, yeasts, their combination, and a water control. After six days, leaf discs were fixed in 100% ethanol and incubated for 3 h under light in 3,3′-diaminobenzidine (DAB)-HCl solution (1 mg/mL, pH 4) following Asselbergh et al. (2007) [70]. All experiments were performed in triplicate.

Leaf discs were photographed after incubation, and disease severity was quantified from the images by automated analysis in R (EBImage, version 4.5.1; R Core Team; R Foundation for Statistical Computing, Vienna, Austria), where necrotic lesion areas were segmented from the disc surface, and the combined area of the two main lesions per disc was expressed as a percentage of the total disc area.

### 2.11. A Field Trial Employing Selected Yeast in Combined Antifungal Strategies

#### 2.11.1. Experimental Vineyard and Study Design

The study was conducted in an experimental vineyard located in Potoče, Vipava Valley, Slovenia. The two grapevine varieties under observation were *V. vinifera* L. cultivars ‘Pinot noir’ and ‘Pinot gris’. The vineyard consisted of 13-year-old vines situated at an altitude of approximately 90 m a.s.l., trained to a single Guyot vertical shoot-positioning system, with a planting density of 5700 vines/ha, and rows oriented in an E-W direction. The experiment was carried out in the 2017 growing season. The experimental design consisted of three vineyard rows for each cultivar, following a randomized plot allocation scheme with five vines per plot and four replicate plots per treatment group. To investigate various combinations of strategies for managing *B. cinerea* infection in vineyards, including canopy management (leaf removal), early/late fungicide (Switch^®^, active ingredients cyprodinil and fludioxonil) application, and biocontrol yeast introduction, the following treatments were applied: Control 1 (C1) = untreated vines (no leaf removal) + 1 × Switch^®^ (early application); Control 2 (C2) = untreated vines (no leaf removal) + 2 × Switch^®^ application; ELR1s = early leaf removal (ELR) + 1 × Switch^®^ (early application); ELR2s = ELR + 2 × Switch^®^ application; ELR1sB = ELR + biocontrol agent in testing + 1 × Switch^®^ (early application); ELR1sK = ELR + commercial biocontrol agent + 1 × Switch^®^ (early application).

The timing of leaf removal was decided based on prior research conducted in the same vineyard on ‘Pinot noir’ [23,24,71]. Pre-flowering (early) leaf removal was performed at the grapevine phenological stage when flowers were separated and inflorescences developed (BBCH 57) by manually detaching the basal four to six leaves per shoot, in accordance with standard pre-bloom defoliation practices [72]. The potential biocontrol agent prepared from the selected strain *P. guilliermondii* ZIM 624 was produced according to Cañamás et al. (2011) [73]. The inoculum was applied under vineyard conditions at a final concentration of 5 × 10^7^ fresh yeast cells/mL.

The commercial biocontrol yeast Botector^®^ (Fertenia S. r. l., Bellizzi, Italy) and the fungicide Switch^®^ (Syngenta AG, Basel, Switzerland) were applied according to the manufacturer’s instructions (0.4 and 0.8 kg/ha, respectively) with Switch^®^ applied either twice (2 s) at grapevine phenological stages BBCH 77 (berries beginning to touch) and BBCH 83 (berries developing colour) or once (1 s) at BBCH 77 (early application). Applications were performed using the standard spray volume and concentration routinely employed by the vineyard manager (1 kg Switch^®^ per 600 L of water). Treatments were applied using a backpack manual sprayer, and special care was taken to avoid spray drift into adjacent plots. The spray volume per plot was approximately 1.1 L.

#### 2.11.2. Visual Examination of Grey Mould

At harvest time (one day before harvesting), the bunches from the experimental plots were visually inspected for signs of infection by *B. cinerea*, as previously described [23]. Infection assessment was conducted directly in the field on the vines by estimating the proportion of affected berries, in accordance with the guidelines of the OEPP (Organisation Européenne et Méditerranéenne pour la Protection des Plantes, 1997) [74]. For this purpose, a group of assessors was trained during the 2010, 2011, and 2015 seasons. *B. cinerea* incidence and severity were calculated for all the plots. Incidence was expressed as the proportion of infected bunches, and severity was calculated as the average infection level in the observed infected bunches.

### 2.12. Statistical Analysis

All statistical analyses were performed using R software (version 4.5.1; R Core Team; R Foundation for Statistical Computing, Vienna, Austria). Statistical analyses and graphical visualizations were performed using the EBImage, FSA, and ggplot2 packages. Differences among treatments were analyzed using one-way analysis of variance (ANOVA). For significant effects, Tukey’s honestly significant difference (HSD) test was applied for multiple comparisons at 95% confidence level (*p* ≤ 0.05). When the assumptions for parametric tests were not met due to high data variability, treatment effects were evaluated using non-parametric Kruskal–Wallis test. Effect sizes were estimated using epsilon-squared (ε^2^), and Dunn-Holm post hoc pairwise comparisons and Wilcoxon test with effect sizes estimated by Cliff’s delta (δ).

## 3. Results and Discussion

### 3.1. Cell Wall-Degrading Enzyme Activities

Yeast-associated chitinases are hydrolytic enzymes capable of degrading chitin, and yeasts producing them have been proposed as putative biocontrol agents (BCA) against various chitin-containing phytopathogens [75,76].

To test the experimental yeast collection for chitinolytic yeasts, a screening protocol designed to detect chromatic changes was employed [49]. By monitoring the chromatic shifts from yellow to purple, our investigation successfully identified several yeast species exhibiting distinctive chitinase enzymatic activities, namely *B. albus, P. guilliermondii*, and *W. anomalus* (Table 1). Additional strain-level details are provided in Table S2. Furthermore, two additional species, *P. kudriavzevii* and *M. pulcherrima*, each showed weak enzymatic activity in one of their tested strains. When evaluated across taxonomic groups, chitinase activity showed differences among genera and families; however, considerable overlap and intra-group variability were observed. These findings suggest that while some taxonomic tendencies may exist, chitinolytic activity is primarily influenced at the strain level (family-level data shown in Figure S1, Supplementary Materials).

The results of our study align with those of the majority of earlier investigations. Previous research by Sugiprihatini et al. (2011) reported chitinolytic activities in both *B. albus* and *P. guilliermondii* [77]. In addition, the ability of *P. guilliermondii* to produce chitinases was documented by Zhang et al. (2011) [56], using the same media as present research with CWP, glucose and sucrose as carbon sources. Chitinase activities were also confirmed in another study in a subset of 11 isolates belonging to *W. anomalus* and *K. ohmeri* [78]. Regarding *M. pulcherrima*, several strains secreting chitinases were previously documented [79] and most recently, Minguet-Lobato et al. (2024) molecularly characterized three new chitinases from the same yeast species [80].

Conflicting findings have been reported for *P. kudriavzevii.* While Madbouly et al. (2020) successfully detected chitinases using an in vitro plate assay [81], Delali et al. (2021) found no evidence of chitinase activities in the four observed *P. kudriavzevii* strains, which is consistent with our results [82].

In the case of *P. membranifaciens,* which was negative for chitinase activity in our study, Lutz et al. (2013) confirmed its chitinolytic activity, but only when the experiments were performed at low temperatures (0 ± 1 °C), whereas the activities were not observed at 20 ± 1 °C [83]. This might still be discouraging because 20 °C is closer to the temperature at which BCA yeasts need to perform in the vineyard environment.

The scientific community has recently shown growing interest in chitinolytic enzymes owing to their diverse potential applications. Although various enzymes may play a role in the breakdown of fungal cell walls, chitinases may play a critical role in biocontrol strategies, as chitin is a key structural element in the cell walls of many fungal plant pathogens [44,84,85]. Fungal chitinases are reported to exhibit inhibitory activities against various pathogenic fungi, including one of the most problematic pathogens in viticulture *B. cinerea* [86,87,88,89]. In addition, these enzymes are capable of lysing even the rigid chitin cell walls of mature hyphae, conidia, chlamydospores, and sclerotia [90].

Following the initial qualitative screening, *P. guilliermondii* ZIM 624 and *W. anomalus* S138 were selected for further investigation.

According to their site of action, chitin-degrading enzymes can be categorized as endo- or exo-acting enzymes [89]. While endo-chitinases randomly facilitate the internal degradation of chitin chains [84,91], exo-chitinases begin their activity at the non-reducing ends of chitin chains, progressively releasing diacetyl chitobiose units [84,92]. To capture potential functional differences, both enzyme activities were quantified separately.

**Figure 1:**
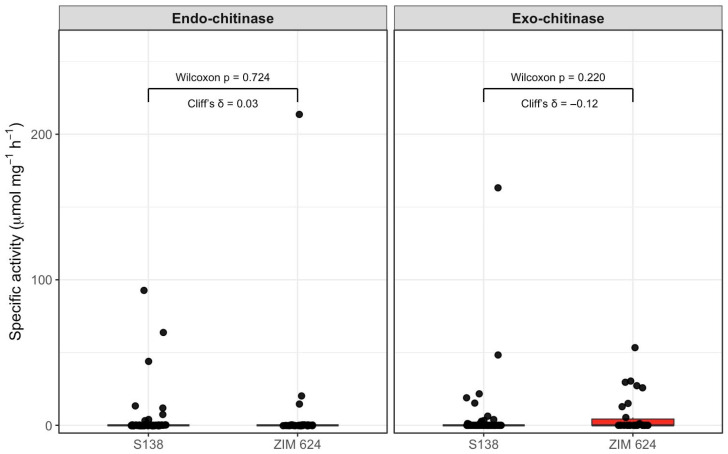
Specific activities of endo- and exo-chitinase (μmol mg^−1^ h^−1^) of *P. guilliermondii* ZIM 624 and *W. anomalus* S138. Data were pooled across culturing incubation times and carbon sources. Points indicate individual measurements; boxplots show median and IQR. Statistical comparisons were performed using a two-sided Wilcoxon test with effect sizes estimated by Cliff’s delta (δ).

Across all tested conditions, endo- and exo-chitinase activities were generally low and highly variable, with many measurements close to or below the analytical detection limit. Although occasional high specific activity values were observed, these occurred sporadically and did not result in consistent strain-specific patterns. When data were pooled across incubation time and carbon source, no statistically significant differences were detected between strains for either endo-chitinase (*p* = 0.724; Cliff’s δ = 0.03) or exo-chitinase activity (*p* = 0.220; Cliff’s δ = −0.12; Figure 1). A visually higher upper range of exo-chitinase activity was observed for *P. guilliermondii* ZIM 624; however, this tendency was weak and driven by a limited number of measurements. Notably, elevated activities were predominantly detected in cultures grown on cell wall preparation (CWP) media at early incubation times (24–48 h), whereas cultures grown on glucose or sucrose generally showed low or non-detectable activity, indicating that chitinase production in both strains is inducible and substrate-dependent rather than constitutively expressed.

Compared with previous reports, the chitinase activities detected in this study were generally lower than those described for *P. guilliermondii* strain M8 under inducing conditions [56] and were consistent with studies reporting low basal chitinase production in *W. anomalus* grown in standard media [78]. Although the statistical support for quantitative differences was weak due to high variability and values close to the detection limit, our data indicate that *P. guilliermondii* ZIM 624 and *W. anomalus* S138 are capable of producing extracellular chitinolytic enzymes in an inducible manner. This is in line with previous observations showing that chitinase expression in antagonistic yeasts is strongly influenced by growth phase, substrate composition, and the presence of fungal cell wall material or hyphae [93,94].

Furthermore, antagonistic yeasts’ capacity to produce extracellular lytic enzymes in response to pathogens may also enhance their adhesion to fungal hyphae, as proposed and corroborated in previous studies [93,95]. This attachment could localize enzyme activity, thereby intensifying pathogen degradation. Subsequent investigations could examine the dual role of our chitinase-positive yeasts: their adherence to *B. cinerea* hyphae and concurrent enzyme secretion. Elucidating this interaction could refine yeast-based biocontrol strategies for more effective fungal suppression in viticulture and beyond.

**Figure 2:**
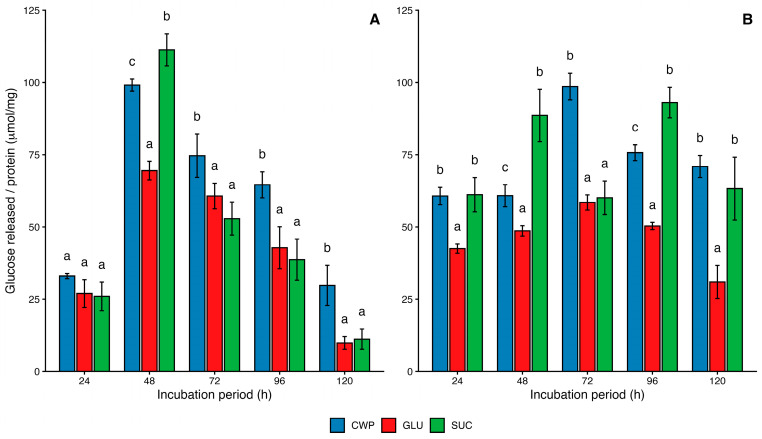
Quantitative characterization of *β*-1,3-glucanase activity in selected yeast strains *P. guilliermondii* ZIM 624 (**A**) and *W. anomalus* S138 (**B**) grown in LB medium supplemented with *B. cinerea* cell wall preparation (CWP), glucose (GLU), or sucrose (SUC) as the sole carbon source for 120 h. Values are expressed as mean ± SD (*n* = 4). Data were analyzed using one-way ANOVA, and means were compared using Tukey’s HSD test (*p* < 0.05). Different letters indicate statistically significant differences among the media at each incubation time.

To gain further insight, two yeast strains, *P. guilliermondii* ZIM 624 and *W. anomalus* S138, which had also been selected for chitinase quantification, were evaluated for *β*-1,3-glucanase activity (Figure 2). *P. guilliermondii* ZIM 624 reached a maximum mean activity of 111.3 U (μmol glucose released mg^−1^ protein h^−1^) on sucrose after 48 h of incubation, whereas *W. anomalus* S138 showed its highest activity of 98.6 U on CWP after 72 h.

After the 48 h peak, *β*-1,3-glucanase activity of *P. guilliermondii* gradually declined on all substrates, with values broadly comparable to those reported by Zhang et al. (2011) for strain *P. guilliermondii* M8, although with a different activity trend [56].

In contrast, *W. anomalus* S138 showed a slower but more sustained increase in activity, peaking between 72 and 96 h, especially on sucrose and CWP. Overall, both strains hydrolyzed multiple carbohydrate sources but exhibited distinct kinetics: *P. guilliermondii* displayed a rapid, short-term response, whereas *W. anomalus* maintained enzyme production for a longer period, making it potentially more suitable for extended fermentations or continuous substrate degradation.

These findings suggest species-specific differences in substrate utilization and the induction of *β*-1,3-glucanase synthesis, likely reflecting the distinct regulatory mechanisms governing enzyme production in the two yeasts.

### 3.2. Glucoside Hydrolase Activities

Yeasts are increasingly explored as biocontrol agents against phytopathogenic fungi in vineyards. At the same time, *β*-glucosidases produced by these yeasts are important enzymes in wine production, as they liberate aromatic compounds from glycosidic precursors and thereby influence wine aroma and flavour. These enzymes can hydrolyse bound terpenes, norisoprenoids, and other volatile molecules during fermentation and ageing, contributing to varietal character and overall sensory complexity [42,96,97]. Moreover, recent study has highlighted the potential of yeast-related *β*-glucosidases to selectively hydrolyze the glycosidic forms of specific phenolic compounds, such as flavonoids, stilbenes, and volatile phenols [56].

In order to evaluate the presence of *β*-glucosidase activities within our yeast collection, we employed a qualitative screening approach [60]. with the omission of potentially inhibitory ferric ammonium citrate.

The distribution of the observed yeast strains capable of producing *β*-glucosidase enzymes is outlined in Table 1, with additional information provided in Table S2.

The results showed that only two of the tested yeast strains belonging to *C. zemplinina* and *S. bayanus* (except on esculin), failed to produce *β*-glucosidase, with *C. zemplinina* being previously reported as unable to produce this enzyme [98]. Most of the remaining strains produced the enzyme, although the activity was weak or variable in *T. delbrueckii* and *K. servazzii* strains.

When evaluated at the family level (Figure S1, upper panel), *β*-glucosidase activity was widely distributed across taxonomic groups, with high proportions of positive strains observed in several families. Although some differences among families were detected, substantial overlap and intra-family variability were evident, indicating that *β*-glucosidase production is broadly present but not strictly conserved at higher taxonomic levels.

These findings align with those documented by Lin et al. (2020) for *T. delbrueckii*, whereas *K. servazzii* showed negative results for all the observed strains in their study (possibly because of the use of ferric ammonium citrate in the media and with only arbutin being tested on YNB agar) [99]. Under their experimental conditions, negative results were also obtained for *W. anomalus* [99]. However, our findings for the *W. anomalus* S138 strain (tested positive) are consistent with those of Lopez-Enriques et al. (2023) [100].

Interestingly, in our investigation some strains belonging to *K. fluxuum*, *K. servazzii*, *L. thermotolerans*, *T. delbrueckii*, as well as *Saccharomyces* and *Pichia* species, tested positive or showed variable results despite these microorganisms are not anticipated to metabolize cellobiose, salicin, or arbutin [101]. But it is worth noting that Fia et al. (2005) also reported the positive growth of *T. delbrueckii* on arbutin and esculin [102].

Concerning *β*-glucosidase activities specifically in *S. cerevisiae*, the current literature reports are inconsistent. Quatrini et al. (2008) [103] conducted a study on an encoding gene within *S. cerevisiae* genome, during which they partially sequenced the *β*-glucosidase gene. Their research revealed that *S. cerevisiae* can secrete *β*-glucosidases [103]. However, Zhang et al. (2021) highlighted in their review that only a few specific *S. cerevisiae* strains exhibit *β*-glucosidase activities and that the same enzyme produced by different yeast strains has different substrate specificities [104]. Nevertheless, several authors have observed considerable *β*-glucosidase synthesis by *S. cerevisiae* [105,106,107] and our study revealed variable strain-dependent outcomes (ranging from mainly positive to weak). Yet, the *S. cerevisiae* Ca1 strain consistently exhibited only weak *β*-glucosidase activity, irrespective of the substrate used.

To clarify whether growth on glucosidic substrates might also reflect the action of *β*-glucan-degrading enzymes, we next assessed *β*-glucanase activity. In the *β*-glucanase assay, all *S. cerevisiae* strains tested positive, with only Ca1 again showing weak activity (Table 1 and Table S2). *β*-glucanase synthesis by *S. cerevisiae* has been previously confirmed [108]. It is also noteworthy that the prior growth observed on glucosidic substrates by some strains of *S. cerevisiae* could have been supported by unspecific exo-acting *β*-glucanases. Even though this claim has not been definitively established, research has demonstrated that exo-*β*-glucanase activities are constitutively expressed, regardless of the carbon source utilized. Initially, this enzyme is found in the periplasmic space, and it is later released into the surrounding culture medium [60,109,110].

Beyond *S. cerevisiae*, most other species in our collection also displayed *β*-glucanase activity, often with pronounced strain-to-strain variability (Table 1 and Table S2; Figure S1). The only tested strains that were devoid of any detectable *β*-glucanase activity in our research were *B. albus* ZIM 608, *C. diversa* ZIM 2110, and some specific strains of *P. kluyveri*, *K servazzii*, *L. thermotolerans* and *P. manshurica* (Table 1 and Table S2). Overall, the results indicate that *β*-glucanase production is widespread among wine-associated yeasts but exhibits considerable intra-species variability, suggesting regulation at the strain level rather than strict taxonomic conservation.

*β*-glucanases, including *β*-1,3-glucanases, play a key role in hydrolysing glycosidic bonds in complex polysaccharides such as glucans and celluloses, generating mannoproteins, oligosaccharides, and glucose and thereby influencing wine stability and sensory properties [111,112,113]. Similar to *β*-glucosidase, they offer numerous potential applications in wine production. In addition, these compounds have been reported to accelerate the composting process of agricultural waste [113] and can be employed in strategies to control yeast spoilage in wine [114,115]. Therefore, a deeper understanding of their functional potentials is crucial.

**Figure 3:**
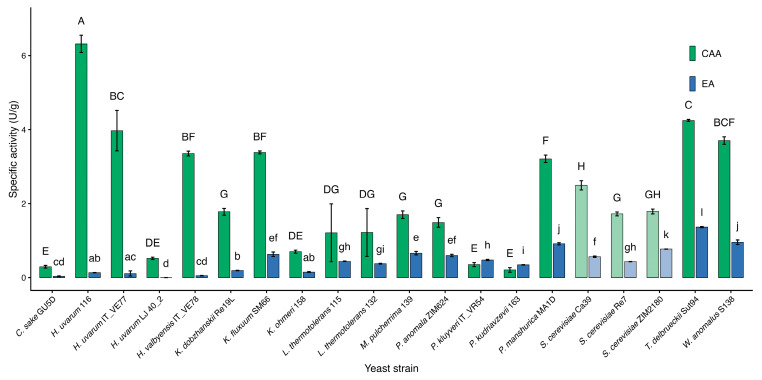
Quantitative assessment of *β*-glucosidase activity on the pNPG substrate in pre-selected *Saccharomyces* (light color shade) and non-*Saccharomyces* yeast strains (dark color shade), showing cell-associated (CAA) and extracellular (EA) activities, expressed as units per gram of dry weight (U/g DW). Values are expressed as mean ± SD (*n* = 4). Data were analyzed separately using one-way ANOVA, and means were compared using Tukey’s HSD test (*p* < 0.05). Different letters indicate statistically significant differences between the strains within each activity.

Contrary to expectations based on physiological classification methods, several yeast species demonstrated an unforeseen ability to assimilate glucosides during pre-screening [101], prompting more detailed investigation. Thus, three *Saccharomyces* and 17 non-*Saccharomyces* strains were selected for further analysis by measuring their *β*-glucosidase activities through quantitative assessment using pNPG (Figure 3).

Yeasts can generate *β*-glucosidase enzymes in diverse cellular locations, such as the cell wall, cytosol, or cell membrane [104,116,117]. While intracellular activities are common, extracellular *β*-glucosidase activities are generally more relevant for industrial applications [104] as intracellular enzymes are released in limited amounts [118]. Nevertheless, Graf et al. (2022) reported that intracellular *β*-glucosidases can serve as robust catalysts under high-pH conditions, as is the case in wine media [117].

Taking this into account, we conducted assays to measure both extracellular specific activities (EA) and cell-associated *β*-glucosidase specific activities (CAA) in the YPD growth medium.

Based on the obtained results, in general, all the tested strains were confirmed to exhibit *β*-glucosidase activities, with *H. uvarum* 116 demonstrating the highest CAA of 6.32 U/g, and *T. delbrueckii* Sut 94 showing the highest EA of 1.36 U/g, both at significant level (Figure 3). Interestingly, the yeast strains examined in our research mainly displayed higher levels of CCA compared to EA. Moreover, a specific *H. uvarum* strain (Lj40.2) exhibited negligible EA while still demonstrating notable CAA.

In the case of *S. cerevisiae*, there are contradictory reports available, with no or variable *β*-glucosidase activities observed in different *S. cerevisiae* strains. Hernandez et al. (2003) [59] studied the *β*-glucosidase activity of two *S. cerevisiae* strains (wild-type and laboratory-originated) using various culture media and glycoside substrates. Although several positive results were obtained, the outcomes varied significantly, depending on the medium or substrate employed [59]. Also, Vernocchi et al. (2011) compared the EA and whole-cell *β*-glucosidase activities of commercial and wild *S. cerevisiae* strains and discovered that the wild strains had significantly higher activities than the commercial strains [119]. Another investigation demonstrated that the observed *S. cerevisiae* strain exhibited notable specific activities in both the EA and CAA domains. Conversely, other strains analyzed in the same study showed a greater tendency towards CAA when compared to their EA levels [60].

In our study, *S. cerevisiae* Ca39 showed the highest average value for CAA (2.5 U/g) among the three selected *S. cerevisiae* strains, whereas EA was relatively low (under 0.75 U/g) for all three strains under observation, with *S. cerevisiae* ZIM 2180 exhibiting a significantly higher value than the other two strains (Figure 3). In a different study, various strains of *Saccharomyces* yeast exhibited varying levels of hydrolase activities when interacting with specific glycosidically bound volatile compounds, and only a limited number of these strains demonstrated real 1,4-*β*-glucosidase activity [60].

In brief, high *β*-glucosidase activity can be considered rare in *S. cerevisiae*, and according to Zhang et al. (2021), only a few strains of *Saccharomyces* exhibit high EA *β*-glucosidase activity [104].

Overall, a variety of studies have shown that yeasts involved in the winemaking process exhibit *β*-glucosidase activity, however, with non-*Saccharomyces* species exhibiting higher levels compared to *S. cerevisiae* [42,96,104,120,121] and that non-*Saccharomyces* yeasts are more likely to produce extracellular glycosidases [122].

Among our tested yeasts, this was true for the majority of non-*Saccharomyces* yeast strains but not for all. The strains that exhibited significantly poorer results for both (exo/endo) activities (compared to selected *S. cerevisiae* yeasts) were *H. uvarum* Lj40.2, *C. sake* Gu5D, and *P. kudriavzevii* 163. Interestingly, *P. kluyveri* IT_VR54 and *P. kudriavzevii* 163 were the only strains with higher average extracellular than cell-associated activities.

As reviewed by Zhang et al. (2021) [104] and Muradova et al. (2023) [123], previous reports on *β*-glucosidase activities are available for a wide range of non-*Saccharomyces* yeasts, including several genera represented also in our collection, such as *Candida*, *Debaryomyces*, *Hanseniaspora*, *Lachancea*, *Kluyveromyces*, *Metschnikowia*, *Pichia*, *Torulaspora*, and *Wickerhamomyces*. Other genera reported to exhibit *β*-glucosidase activity include *Brettanomyces*, *Dekkera*, *Hansenula*, *Rhodotorula*, *Saccharomycodes*, *Schizosaccharomyces*, and *Zygosaccharomyces*.

Nevertheless, the reported results, as well as our findings, are frequently strain-specific, underscoring the importance of thoroughly examining each yeast strain potentially involved in the wine production chain.

In addition to laboratory studies of enzymatic activities, a variety of non-*Saccharomyces* yeasts with good *β*-glucosidase activities have also been tested in real-world winemaking scenarios. Promising outcomes have been reported for wine aromatic improvements owing to the liberation of volatile compounds from their precursors, including higher alcohols, terpenes, and esters. Testa et al. (2020) successfully introduced *H. guilliermondii* BF1 into the fermentation process of Fiano wine, resulting in higher linalool and terpene-4-ol contents with enhanced fruity and rose aroma [124]. Non-*Saccharomyces* yeast strains belonging to *I. terricola, P. kudriavzevii* and *M. pulcherrima* were tested by Qin et al. (2021) in Cabernet Sauvignon and evolved lower content of C6 compounds, benzene derivative, and fatty acid ethyl ester compounds and higher content of terpene, *β*-ionone, higher alcohol, and acetate compounds in comparison to *S. cerevisiae* [125]. In addition, *H. uvarum* is often reported to have high *β*-glucosidase activity and effective application in winemaking [104,126]. Furthermore, terpen hydrolysis in Muscat wine was studied after treatment with immobilized commercial *β*-glucosidase, and aroma liberation was observed, resulting in wines with high concentrations of nerol and geraniol [125,126]. Gao et al. (2022) [96] studied purified *β*-glucosidases from two non-*Saccharomyces* strains, *M. guilliermondii* NM218 and *H. uvarum* BF345, both of which exhibited good performance in Chardonnay wine fermentation and aging by contributing esters, terpenes, C13-norisoprenoids, higher alcohols, and fatty acids to the wine. Interestingly, *β*-glucosidase from *M. guilliermondii* strain imparted sweet, floral, fruity, and banana aromas, whereas *β*-glucosidase from *H. uvarum* strain also contributed honey and pomelo aromas to wine [96].

Finally, it is important to note that, although several non-*Saccharomyces* yeast strains observed in our study exhibited glycosidase activity, these results indicate potential rather than actual performance. Although vinification trials were beyond the scope of this study, the findings provide a basis for future investigations under realistic winemaking conditions, where enzyme activity may be influenced by the harsh conditions of the wine matrix, including acidity and ethanol content.

### 3.3. β-Lyase Activity

Yeast-associated *β*-lyase activity during vinification may liberate volatile sulfur compounds called thiols, which are detectable at very low concentrations [127,128]. They are often associated with pleasant tropical fruit flavors in wines, with descriptors commonly including passion fruit, grapefruit, and guava [127].

There are two cellular metabolic enzymes, cysteine-S-conjugate *β*-lyase and cystathionine *β*-lyase, reported to be involved in sulfur-containing amino acid biosynthesis. They can catalyze *β*-elimination reactions with cysteine-S-conjugates and, occasionally, with aliphatic and aromatic substitutions [129].

To assess *β*-lyase activities, yeast strains were grown in YCB–SMC selective medium containing S-methyl-L-cysteine (SMC), which mimics grape aroma precursors and releases ammonium upon cleavage, thereby enhancing yeast growth and colony size.

Our findings are summarized in Table 1, with Table S2 providing more detailed information on each yeast strain. The results revealed that *β*-lyase activity was extensively distributed and observed in most of the yeasts examined. *β*-lyase activity was widely distributed across the tested yeast collection, with most strains showing positive growth on YCB–SMC medium (Table 1 and Table S2; Figure S1).

In the case of *Saccharomyces* species, *S. cerevisiae* and *S. paradoxus* both tested positive for all but one strain (weak). *S. kudriavzevii* was positive for all seven strains, whereas *S*. *bayanus* tested negative. Several researchers have previously noted that the activity of *β*-lyase and/or the ability of *S. cerevisiae* to produce volatile thiols is a trait that varies among strains [45,130,131,132]. Cordente et al. (2022) reported up to a nine-fold variation in cysteine-S-conjugate *β*-lyase activity among 39 *S. cerevisiae* wine strains [132]. And the most recent study by Breia et al. (2025) focused on autochthonous yeasts, also reported pronounced strain-dependent variability as well as lack of clear distinction between *S. cerevisiae* and non-*Saccharomyces* species [133].

In addition to *S*. *bayanus,* some non-*Saccharomyces* yeasts (*C. diversa*, *C. oleophila*, *C. vini*, and *C. zemplinina*) tested negative in our study (Table 1). Furthermore, *K. servazzii*, *L. thermotolerans*, *P. kudriavzevi*, *H. uvarum* and *P. manshurica* showed variable results.

However, it is worth noting that *M. manshurica* was mainly positive (13/14). The majority of the others (excluding some not determined) displayed positive outcomes. Overall, the observed variability supports previous reports indicating that *β*-lyase activity is largely strain-specific rather than strictly species-dependent.

Regarding positive non-*Saccharomyces* yeasts, our research findings are in line with the results of a recent study conducted by López-Enríquez et al. (2023), who highlighted the significance of *T. delbrueckii* isolates owing to their substantial *β*-lyase activities, as well as *P. kudriavzevii*, which exhibited positive but unique enzymatic activities in all 13 isolates [100]. The same authors also reported *β*-lyase activity in *W. anomalus* strains. In addition, Belda et al. (2016) documented widespread *β*-lyase activity in their analysis of 770 yeast isolates of diverse enological origins [46]. However, in their study, moderate activity was observed more frequently than strong activity, and the results for most species were variable, with only *T. delbrueckii*, *M. guilliermondii*, and *K. marxianus* exhibiting overall positive specific behaviors [46]. The former two displayed similar behaviors in our investigation. Nevertheless, the latter species was not included in our research.

Volatile thiols originate from non-aromatic (bound) precursors in grapes, which are converted during fermentation through yeast *β*-lyase activity. Yeast strains with higher *β*-lyase activity are therefore expected to be more effective in releasing these volatile compounds into wine. To explore this potential in more detail, we analyzed the protein extract activity of ten pre-selected *β*-lyase-positive strains using cysteine as a physiological substrate. Because cysteine conjugates are costly and scarce, an indirect assay was employed, measuring pyruvate released during cysteine conversion. Pyruvate was enzymatically reduced to lactate by L-lactate dehydrogenase, with concurrent NADH oxidation; the decrease in absorbance at 340 nm reflected the cysteine-*β*-lyase activity [61].

**Figure 4:**
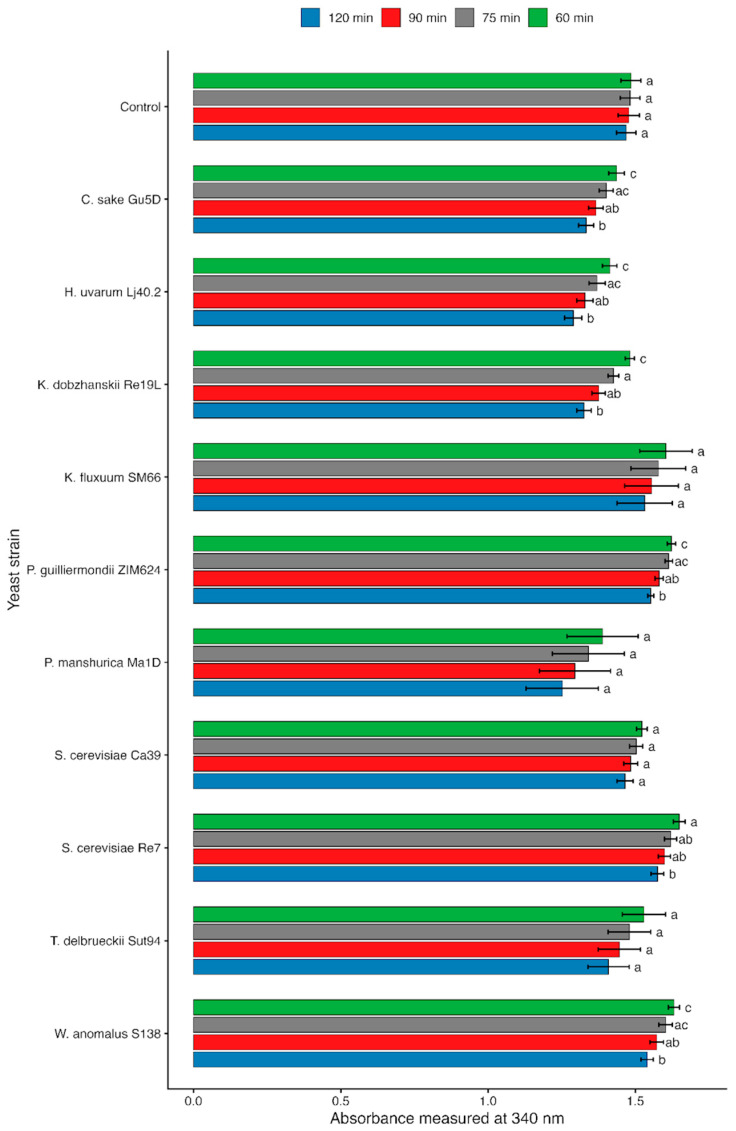
Cysteine *β*-lyase activity of ten pre-tested *Saccharomyces* (2) and non-*Saccharomyces* (8) yeast strains. Values are expressed as mean ± SD (*n* = 3). Data were analyzed by one-way ANOVA, and means were compared using Tukey’s HSD test (*p* < 0.05). Different letters indicate statistically significant differences among reaction times (60, 75, 90 and 120 min) during assay progression within each individual strain.

After adding L-lactic dehydrogenase and NADH to the reactions, the A340 measurement at 60 and 75 min showed immediate significant NADH consumption just in case of *K. dobzhanskii* Re19L (Figure 4). For *P. guilliermondii* ZIM 624, *W. anomalus* S138, and *S. cerevisiae* Re7, the NADH concentration was slightly higher compared to control, probably due to transfer of some NADH from the yeast cell. With the proceeding of the reaction up to 120 min, the NADH concentration dropped significantly in comparison with situation at 60 min for the following yeasts: *S. cerevisiae* Re7, *S. cerevisiae* Ca39, *C. sake* Gu5D, *H. uvarum* Lj40.2, *K. dobzhanskii* Re19L, *P. guilliermondii*, and *W. anomalus* S138 (Figure 4). The remaining strains exhibited promising trends, although with considerable deviations.

Given the significance of various thiols in the aromatic profile of mainly white, but also red wines [132,134], the research has expanded beyond enzyme activity assays to include the actual release of thiols during fermentation with diverse yeasts and grape varieties. The three most investigated volatile thiols that can significantly contribute to a specific fruity aroma are 3MH (3-mercaptohexan-1-ol), 3MHA (3-mercaptohexyl acetate), and 4MMP (4-mercapto-4-methylpentan-2-one) [128,134].

Several authors reported the ability of *S. cerevisiae* to release varietal thiols under vinification conditions with notable variation reported among strains [61,130,131,135]. The available data are relatively scarce for non-*Saccharomyces* strains; however, the findings of Anfang et al. (2009) [136] suggest that co-fermentation with two isolates of *P. kluyveri* could potentially enhance the concentration of 3MHA in Sauvignon blanc. Moreover, two isolates of *C. zemplinina* yielded considerable amounts of 3MH [136]. Another study discovered that particular strains of *T. delbrueckii* and *M. pulcherrima* demonstrated the ability to liberate 3SH in Sauvignon Blanc wines; however, they exhibited limited capacity for releasing 4MSP [137]. Furthermore, a strain of *M. pulcherrima* was found to affect 4-MSP levels when co-fermented with *Saccharomyces cerevisiae* [43].

Results of the cysteine *β*-lyase assay suggest that several indigenous yeasts exhibit enzymatic features consistent with a potential contribution to varietal thiol release during vinification. Cysteine *β*-lyase activity was assessed in vitro using cysteine as a physiological substrate, with NADH depletion used as an indirect indicator of enzymatic activity. *K. dobzhanskii* Re19L showed the most rapid and pronounced NADH consumption, while *P. guilliermondii* ZIM624, *W. anomalus* S138, and *S. cerevisiae* strains Re7 and Ca39 also exhibited time-dependent NADH depletion, indicating detectable *β*-lyase activity (Figure 4).

Based on these screening results, selected *β*-lyase–positive strains (*K. dobzhanskii* Re19L and *P. guilliermondii* ZIM 624) were subsequently evaluated in separate vinification experiments reported as a conference communication [138], where they were included in sequential fermentations of Sauvignon Blanc and Istrian Malvasia musts. In that study, fermentations involving these strains were associated with increased concentrations of key varietal thiols (3MH, 3MHA, 4MMP and BM) relative to reference fermentations, consistent with an enhanced capacity to contribute to thiol release.

Formation of 3MHA likely reflected not only *β*-lyase–mediated release of 3MH but also the involvement of downstream yeast enzymes, such as alcohol acyltransferases. In this context, *K. dobzhanskii* Re19L was associated with higher 3MHA levels and enhanced perception of fresh tropical aromas, and ranked highest in the sensory evaluation of Sauvignon Blanc wines [138].

Overall, these findings indicate that cysteine *β*-lyase activity represents a useful screening criterion for identifying thiol-releasing starter cultures; however, the present work evaluates enzymatic potential under laboratory conditions only, providing a basis for future vinification trials.

### 3.4. Sulfite Reductase Activity and H_2_S Production

In winemaking, the yeast-related formation of hydrogen sulfide is considered unfavorable, as it is linked to an unpleasant aroma referred to as “rotten egg” odor [132,139,140]. The presence of H_2_S in wine is particularly problematic because of its low sensory threshold, which has been established as 1.1 μg/L for red wine and 1.6 μg/L for white wine [141,142]. H_2_S is produced in wine as an outcome of yeast metabolic activities through various mechanisms and sources [143], with sulfite reductase being the primary enzyme responsible for catalyzing the relevant reactions [140,144].

Therefore, we assessed H_2_S production resulting from sulfite reductase activity. In our investigation, *P. manshurica* strain Sut 91.2 demonstrated notable hydrogen sulfide generation, whereas strain M49, belonging to the same species, showed only weak H_2_S presence, and the other 12 observed *P. manshurica* strains showed no presence at all (Table 1 and Table S2). Although data on this species remain limited, recent studies by Tzamourani et al. (2023) revealed that *P. manshurica* can produce H_2_S [145]. Interestingly, in their screening conducted in Greece, it was also observed that spontaneously fermented wine isolates from the regions of Nemea and Pelion exhibited lower levels of H_2_S than those isolated from two other regions, regardless of the species parameter [145], highlighting the importance of location-related specifics.

Among the examined non-*Saccharomyces* yeasts (apart from the aforementioned *P. manshurica* strains), solely *H. uvarum* ZIM 670 and *L. thermotolerans* Re18_D demonstrated positive outcomes, albeit only as weak producers (Table 1). This finding aligns with Belda et al. (2016) [46], who discussed that H_2_S production from sulfite reductase activity is generally infrequent in non-*Saccharomyces* species. In their study only several *H. uvarum, H. osmophila, H. opuntiae*, and *T. delbrueckii* isolates showed notably high H_2_S production. Contrary to this prevailing notion, numerous studies have reported H_2_S production in various non-*Saccharomyces* species, although with significant variations across different investigations. A study conducted by Lin et al. (2020) [99] focused on 77 indigenous non-*Saccharomyces* isolates comprising seven species from five genera: *Kazachstania, Aureobasidium*, *Meyerozyma*, *Wickerhamomyces*, and *Torulaspora*. Their results (Biggy) indicated that all the observed isolates, except one, were capable of producing H_2_S [99]. Furthermore, Ge et al. (2023) [146] evaluated six species from four genera (*Hanseniaspora*, *Saccharomyces*, *Rhodotorula*, and *Metschnikowia*) for their sulfite reductase enzymatic activities with variable results observed also in their study namely for *Hanseniaspora* and *Rhodotorula* species, displaying either no or low production of sulfite reductase. In addition, in their study, only one strain of *Metschnikowia* yeast produced a low level of H_2_S [146].

In the case of *Saccharomyces* species, in our study, *S. paradoxus* strains tested negative, *S. cerevisiae* strains demonstrated negative to weak (one strain) outcomes, and *S. bayanus* was characterized as a weak producer (Table 1).

Contrary to some of our findings, Sipiczki et al. (2001) isolated several *Saccharomyces* yeasts from Tokay wine made from botrytized grapes and characterized *S. bayanus* together with *S. paradoxus* and *S. cervisiae* as strong H_2_S producers [147]. Other reports have described strain-dependent variability among *S. cerevisiae* isolates [45,145]. Moreover, Ge et al. (2023) confirmed sulfite reductase activity in all screened *S. cerevisiae* strains, although some exhibited only low production levels [146].

These more recent studies all corroborate previous findings that *Saccharomyces* yeasts can significantly influence the formation of H_2_S-associated off-flavors; however, the occurrence of this off-flavor in wine is highly dependent on the specific yeast strain selected for fermentation [148,149,150].

### 3.5. In Vitro Yeast-Mediated Fungal Mycelial Radial Growth Inhibition (Y-FMGRI)

**Figure 5:**
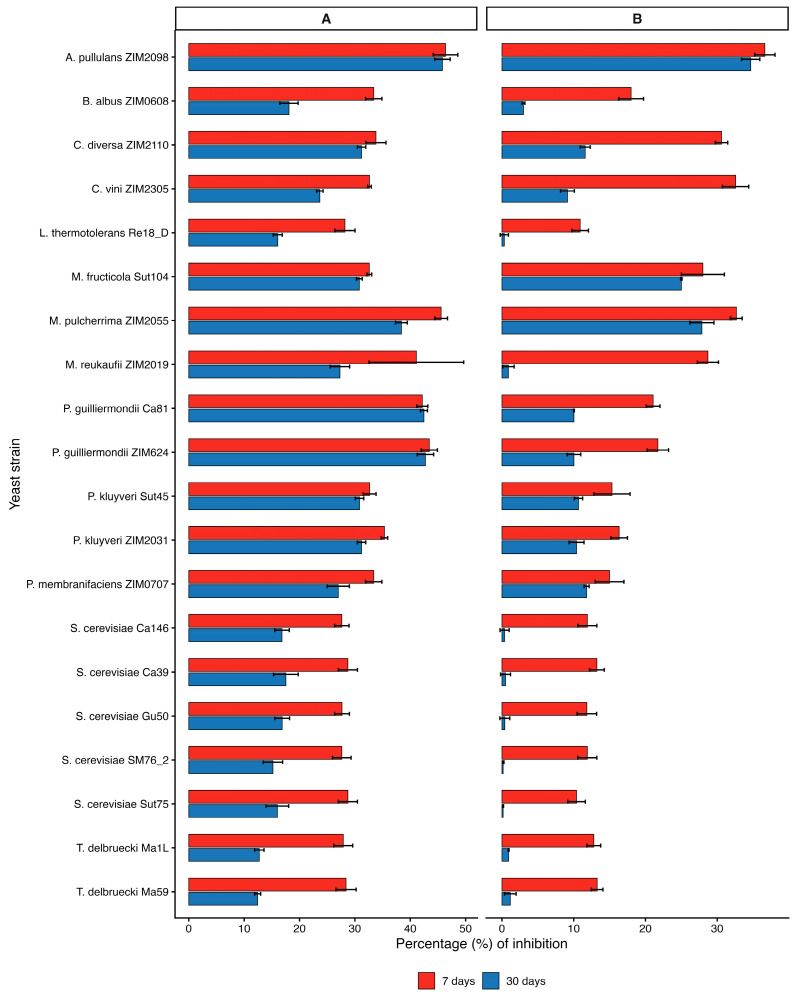
Yeast-mediated inhibition of fungal mycelial radial growth (Y-FMRGI), showing the 20 best inhibitory outcomes on grape juice medium—GJM (**A**) and yeast extract–peptone–dextrose agar—YPDA (**B**) after 7 and 30 days. Values are expressed as mean ± SD (*n* = 3). Statistical analyses for all individual strains were performed using the non-parametric Kruskal–Wallis test with Dunn-Holm post hoc test and are provided in Supplementary Materials, Tables S3 and S4.

The results of testing for Y-FMGRI, as presented for the best outcomes in Figure 5 and summarized in Tables S3 and S4, indicate that strains *A. pullulans* ZIM 2098, *M. pulcherrima* ZIM 2055, *M. reukaufii* ZIM 2019, *P. guilliermondii* ZIM 624 and Ca81 ranked among the yeasts with the highest average inhibitory activity against *B. cinerea* on grape juice medium (GJM) after seven days, with inhibition values ranging from 42.1 to 45.4%. In addition, several yeasts, namely *B. albus*, *C. diversa*, *C. vini*, *M. fructicola*, and both strains of *P. kluyveri*, demonstrated noteworthy average inhibition of fungal growth (>30%). In cases where multiple strains within a species were examined, differences at the species level appeared more pronounced than differences among strains, based on observed inhibition trends.

Compared to GJM, after seven days on YPDA, *A. pullulans*, *C. vini*, *M. pulcherrima*, and *C. diversa* demonstrated the highest average inhibition percentages, ranging from 30.6% to 36.6%. These values were consistently lower than those observed on GJM, indicating a medium-dependent trend in inhibitory performance. This difference is likely attributable to the distinct chemical composition of GJM, which reflects real grape juice and contains a complex mixture of sugars, organic acids, phenolics, nitrogenous compounds, aromatic substances, vitamins, minerals, and pectins. Such nutrient richness may enhance yeast competitiveness, suggesting that competition for nutrients could contribute to yeast-mediated biocontrol activity [151].

In comparison with the results of other studies, recent research conducted by Flores et al. (2025) may further indicate the medium-dependent nature of yeast antagonism [35]. The authors employed Czapek agar to evaluate grape-associated yeast strains, including those from the genera *Saccharomyces*, *Torulaspora*, *Debaryomyces*, and *Candida*, against four *B. cinerea* strains. Their findings revealed considerable variability in the inhibitory effectiveness among *S. cerevisiae* isolates. The highest average inhibition rate (52.3%) was observed for *S. cerevisiae* BSc14 against the *B. cinerea* strain B24, whereas some other strains also exhibited relatively high inhibition levels ranging from 24.0 to 51.6% [35]. These reported inhibition levels are higher than the median observed for *S. cerevisiae* strains on both media and at both observation times (7 and 30 days) in the present study.

In contrast, the two *T. delbrueckii* strains examined in the present study exhibited markedly higher inhibitory efficiency on both media than those reported by Flores et al. (2025) [35]. It is also noteworthy that none of the 18 strains evaluated in their study demonstrated effective inhibition against all four *B. cinerea* strains tested, emphasizing the strong dependence of biocontrol efficacy on yeast and pathogen strain identity, as well as on assay conditions, particularly the growth medium.

When analyzed across taxonomic groups (Figure S2), Y-FMGRI inhibition differed significantly among families on both media (Kruskal–Wallis, *p* < 0.001). Nevertheless, substantial overlap in inhibition values and pronounced intra-family variability were observed. Strong inhibitory phenotypes occurred across multiple families rather than being restricted to a single lineage. This pattern indicates that antagonistic performance is not strictly conserved at higher taxonomic levels but is largely strain-dependent. A similar trend was observed at the genus level, where significant overall differences were detected, yet broad variability within genera remained evident.

This is further supported by a study by Maluleke et al. (2022), who evaluated yeast antagonistic activity on MEA and low-glucose (0.2% *w*/*v*) YPD agar (YPD-L) [152]. The assay included *A. pullulans*, *C. oleophila*, *P. guilliermondii*, *P. kluyveri*, and *P. manshurica* species that were also examined in the present study. Inhibition levels varied widely among species: *A. pullulans* showed 39.22% inhibition, *C. oleophila* ranged from no detectable inhibition to 32.02%, and *P. guilliermondii* reached 44.99% [152].

These values are similar to those obtained on GJM in our study; however, in contrast to Maluleke et al. (2022) [152], inhibitory activity was also detected in *P. kluyveri* and *P. manshurica* strains, which showed no detectable inhibition under their assay conditions (Tables S3 and S4).

Additional evidence for the strong medium dependence is provided by a direct comparison with the study by Raspor et al. (2010) [48]. Their study evaluated four yeast strains (ZIM 2098, ZIM 2055, ZIM 2019, and ZIM 624), which were also examined in our research, and reported higher inhibition levels than those observed in the present study. However, Raspor et al. (2010) [48] were using NYDA medium.

To the best of our knowledge, yeast-mediated biocontrol activity has not been previously evaluated using a diluted natural grape juice medium designed to better approximate a realistic grape berry environment.

### 3.6. Inhibitory Activity of Yeast-Modulated Antifungal Volatile Organic Compounds (VOCs)

**Figure 6:**
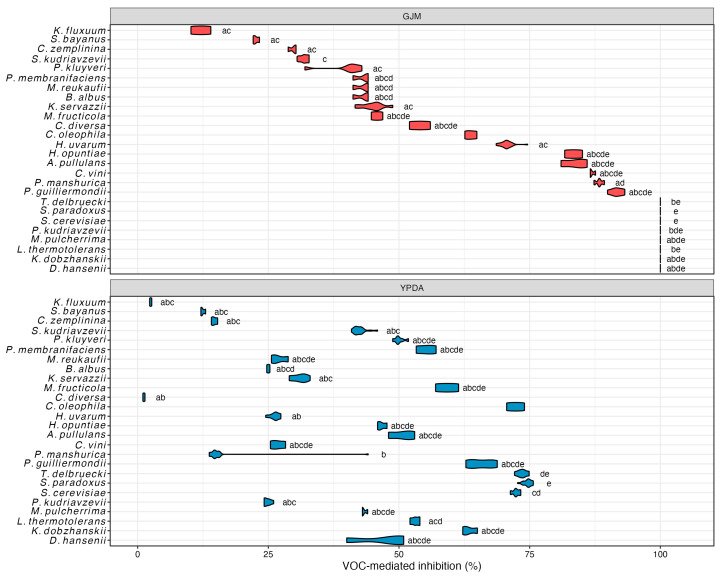
VOC-mediated inhibition (%) of *B. cinerea* F61 by yeast species grown on grape juice medium (GJM) and YPDA. Species are ordered by median inhibition on GJM (top to bottom), and the same order is maintained in the YPDA panel for comparison. Violin plots represent the distribution of replicate measurements. Letters indicate statistically homogeneous groups within each medium based on Dunn–Holm adjusted pairwise comparisons (α = 0.05); species sharing at least one letter are not significantly different.

The inhibitory effects of yeast-modulated VOCs against *B. cinerea* germination were evaluated using a double Petri dish system. Figure 6 and Tables S5 and S6 present the summarized median inhibition values and pairwise statistical comparisons.

VOC-mediated inhibition was detected across most tested yeasts, and generally species-level differences were observed. Inhibition levels were strongly dependent on the growth medium used for VOC production.

On GJM, several yeast species showed very high VOC-mediated inhibition, frequently reaching almost complete inhibition (median ≥ 95%). This pattern was observed for *D. hansenii*, *K. dobzhanskii*, *M. pulcherrima*, *L. thermotolerans*, *P. kudriavzevii*, *S. cerevisiae*, *S. paradoxus* and *T. delbrueckii*. High inhibition (median 80–95%) was also consistently observed for *A. pullulans*, *C. vini*, *H. opuntiae*, *P. guilliermondii* and *P. manshurica*.

Lower inhibition was recorded for *K. fluxuum* (median ≈ 12%), *S. bayanus* (≈23%) and *C. zemplinina* (≈30%). Although global Kruskal–Wallis tests indicated large medium- and species-level effects, Dunn–Holm–adjusted pairwise comparisons among yeasts were mostly non-significant, reflecting high within-group variability and limited statistical power (Tables S5 and S6).

VOC-mediated inhibition on YPDA was generally lower than on GJM. The highest inhibition values (median > 70%) were observed for *C. oleophila*, *S. cerevisiae*, *S. paradoxus* and *T. delbrueckii*, whereas *C. diversa* and *K. fluxuum* showed minimal inhibition.

In contrast to the general trend, *C. oleophila*, *M. fructicola*, *P. kluyveri* and *S. kudriavzevii* exhibited higher inhibition on YPDA than on GJM, indicating species-specific responses to medium composition.

When analyzed across taxonomic groups (Figure S3), VOC-mediated inhibition differed significantly among families on both media (GJM: Kruskal–Wallis H = 31.64, *p* < 0.001; YPDA: H = 52.38, *p* < 0.001). Nevertheless, substantial overlap among families and pronounced intra-family variability were observed. Strong inhibitory phenotypes occurred across multiple lineages, particularly within *Saccharomycetaceae*, *Debaryomycetaceae* and *Metschnikowiaceae*, rather than being restricted to a single taxonomic group. A comparable pattern was evident at the genus level (visualization not shown), where significant overall differences were detected but high within-genus variability remained apparent. These results indicate that although certain taxonomic tendencies may exist, VOC-mediated antagonism is predominantly strain-dependent rather than strictly conserved at higher phylogenetic levels.

Several studies have reported antifungal activity of yeast-emitted VOCs against *B. cinerea*, although direct comparison of inhibition levels across publications is often limited by differences in experimental design and selected endpoints. In the study by Flores et al. (2025), VOC-mediated antagonism was assessed as reduced mycelial growth of four *B. cinerea* strains [35]. In that study, 13 of 18 yeast isolates produced volatiles that significantly inhibited at least one *Botrytis* strain, with inhibition values ranging from 21 to 77%, depending on the yeast–pathogen combination. VOCs produced by *S. cerevisiae* BSc206 significantly inhibited the mycelial growth of the three *B. cinerea* strains, with inhibition values between 26% and 55% [35].

Cordero-Bueso et al. (2017) reported inhibition of *B. cinerea* by VOCs emitted by *H. uvarum*, *Metschnikowia* spp., *M. guilliermondii* (syn. *P. guilliermondii*), and *S. cerevisiae* as a frequent producer of inhibitory volatiles [17]. Similarly, Nally et al. (2015) showed that volatiles emitted by both *Saccharomyces* and non-*Saccharomyces* yeasts possess antifungal activity, although the inhibition levels were generally moderate and dependent on assay conditions, yeast strain, and growth substrate [34]. Thus, the literature indicate that VOC-mediated antagonism is widespread among yeasts and our results are consistent at the species level, by identifying *S. cerevisiae*, *T. delbrueckii*, *M. pulcherrima*, *A. pullulans* and *P. guilliermondii* as strong VOC-mediated antagonists of *B. cinerea*. The relevance of *P. guilliermondii* is further supported by our recently published study [36], in which 65 VOCs emitted by *P. guilliermondii* ZIM 624 were assessed using HS-SPME–GC–MS. Several compounds—particularly monoterpenes (citronellol, geraniol, nerol, linalool and α-terpineol), as well as 4-vinylphenol and isoamyl acetate—showed strong inhibitory activity against *B. cinerea* mycelial growth in fumigation assays. Moreover, the VOCs produced by *P. guilliermondii* ZIM 624 significantly reduced gray mould incidence and disease severity in grape berries, supporting the relevance of VOC-mediated antagonism [36].

In our experimental design, yeasts were pre-grown for two days before assembling the double-dish assay with the *B. cinerea*–inoculated plate, allowing yeast biomass accumulation and volatile production prior to fungal exposure. The differences from studies in which yeasts and *B. cinerea* were inoculated simultaneously may partly explain the variation in the reported inhibition levels. Despite the quantitative variability among studies, the available evidence supports VOC emissions as a biologically relevant antagonistic trait among vineyard-associated yeasts.

### 3.7. Yeast Tolerance to Copper and Commercial Fungicides

Numerous yeast species exhibit inherent insensitivity to antifungal agents commonly used in vineyards; therefore, yeast strains tolerant to copper and tested fungicides may be of particular value in biocontrol applications [39,153].

**Figure 7:**
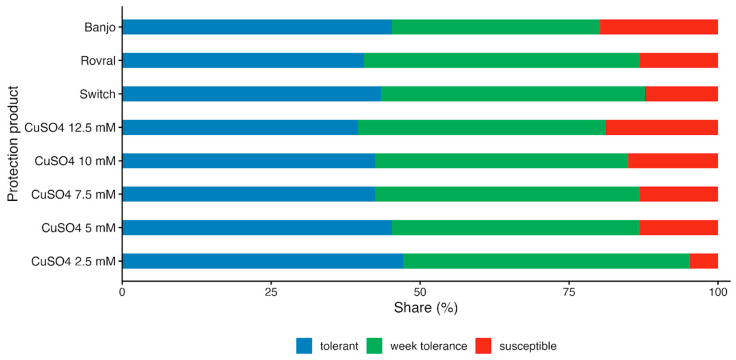
Percentage distribution of yeast strain tolerance categories in response to copper sulfate (CuSO_4_) at increasing concentrations and three commonly used vineyard fungicides, based on qualitative growth response. Values are expressed as percentages of the total number of strains tested for each treatment. Detailed results for each individual strain are provided in Supplementary Materials, Table S7.

Figure 7 indicates that across all tested commercial fungicides and copper concentrations, most yeast strains exhibited either full or weak tolerance, whereas susceptibility accounted for a smaller proportion of the responses. This pattern suggests the high resilience of vineyard-associated yeast to commonly used disease control products.

At the strain level, 32 strains exhibited high tolerance to all tested copper concentrations and to all three fungicides under observation (Table S7). These included *A. pullulans*, *H. opuntiae*, *H. uvarum* (7 strains), *K. dobzhanskii*, *K. fluxuum*, *L. thermotolerans* (7 strains), *M. reukaufii*, *P. guilliermondii* (2 strains), *P. kluyveri* (2 strains), *P. manshurica* (9 strains), *P. membranifaciens*, and *S. cerevisiae* (2 strains), *S. bayanus*, *S. cerevisiae* (1 strain). In contrast, four strains, namely *K. servazzii* F48, *S. paradoxus* Gu95, M22, and Sut103, showed no tolerance to any of the tested antifungal agents. Notable strain-specific characteristics were also observed (Table S7).

*A. pullulans* has been previously reported to exhibit copper tolerance [154,155]. In addition, *H. uvarum* displays high tolerance to copper [38,155]. Grangeteau et al. (2017) further reported copper tolerance in several other yeast species, including *H. guilliermondii*, *Metschnikowia* spp., and *P. membranifaciens* [155]. Yeast tolerance to copper-based fungicides appears to be strongly influenced by the ecological origin of the isolates [38,156]. Vineyard-associated strains of *S. cerevisiae* commonly exhibit elevated copper tolerance, which has been linked to the tandem amplification of the metallothionein gene, *CUP1* [157]. Accordingly, copper resistance is regarded as a vineyard-related adaptation, as it is predominantly observed in vineyard strains of *S. cerevisiae*, whereas *S. paradoxus* and wild *S. cerevisiae* strains are generally copper-sensitive [38,155]. Supporting the importance of ecological origin, Vadkertiová and Sláviková (2006) reported that *S. cerevisiae* strains isolated from diverse non-vineyard environments, such as water, soil, and tree leaves, were sensitive and unable to tolerate even low copper concentrations [156].

Consistent with these observations, in the present study, most *S. cerevisiae* strains exhibited at least weak tolerance to copper, whereas *S. paradoxus* strains showed no or weak resistance (Table S7).

Interestingly, several strains that exhibited high tolerance to all tested copper concentrations in our study were not directly sourced from vineyards but were collected from the nearby areas. Specifically, these strains include *L. thermotolerance* SV34 and V6, *P. manshurica* SV37D and SV37L (soil under an oak tree), *L. thermotolerance* SV33 (oak bark), and *H. uvarum* Sut80 (oak leaves and twigs, collected near the vineyard).

While organic viticulture is largely limited to copper- (and sulfur-) based products, conventional systems employ a broader range of preventive and curative synthetic fungicides [158]. The most frequently used fungicides in the Vipava Valley winegrowing area were evaluated: Switch^®^ (active compounds cyprodinil and fludioxonil), Rovral^TM^ (active compound iprodione), and Banjo^®^ (active compound fluazinam).

The results showed widespread tolerance with *A. pullulans*, *D. hansenii*, *H. opuntiae*, *H. uvarum* (7 strains), *K. dobzhanskii*, *K. fluxuum*, *L. thermotolerans* (7 strains), *M. reukaufii*, *P. guilliermondii* (2 strains), *P. kluyveri* (2 strains), *P. manshurica* (11 strains), and *S. cerevisiae* (4 strains), demonstrating strong resistance to all three tested fungicides (Table S7). Overall, a higher proportion of sensitive responses was observed for Banjo^®^, whereas Switch^®^ and Rovral^TM^ showed similar sensitivity patterns (Figure 7).

Using a different set of commonly used fungicides, Magoye et al. (2020) likewise reported widespread insensitivity among naturally occurring yeasts and highlighted the notable fungicide tolerance of selected *A. pullulans* isolates [39]. Given the wide range of fungicides used in vineyards, the practical biocontrol potential of yeast strains should ultimately be pre-assessed against those most relevant to the site of the intended application.

### 3.8. Niche Overlap Index (NOI)

To evaluate potential inhibition via competition for nutrients, niche similarity among 20 pre-selected yeast species was assessed by comparing the in vitro utilization profiles of 14 grape-related carbon resources and calculating the NOI.

**Figure 8:**
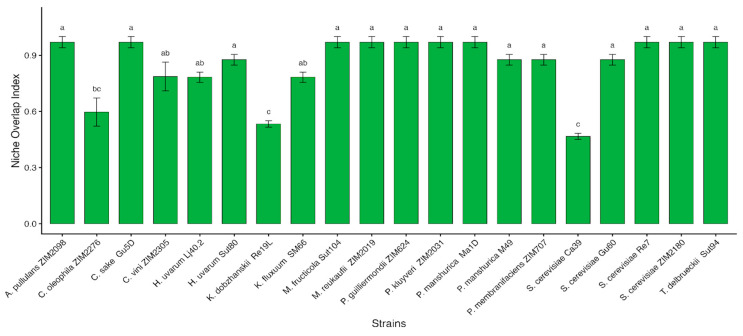
Niche overlap index (NOI) values among pre-selected *Saccharomyces* and non-*Saccharomyces* yeast strains based on carbon source utilization profiles. NOI values > 0.90 indicate the occupation of the same niche (competitive exclusion). Values are expressed as mean ± SD (*n* = 3). Data were analysed by one-way ANOVA, and means were compared using Tukey’s HSD test (*p* < 0.05). Different letters indicate statistically significant differences among strains.

Ten yeast–fungus interactions indicated strong niche overlap and competition for shared nutrient resources (NOI > 0.90) (Figure 8). These outcomes involved strains able to utilize all 14 tested carbon sources and included selected strains of *A. pullulans*, *C. sake*, *M. fructicola*, *M. reukaufii*, *P. guilliermondii*, *P. kluyveri*, *P. manshurica*, *T. delbrueckii* and *S. cerevisiae*. Four additional strains showed average NOI values > 0.85, belonging to *H. uvarum*, *P. manshurica*, *P. membranifaciens*, and *S. cerevisiae*. *S. cerevisiae* Ca39 and *K. dobzhanskii* Re19L showed significantly lower NOI compared to the other observed strains.

In a previous study Nally et al. (2015) reported six *Saccharomyces*–fungus and nine non-*Saccharomyces*–fungus interactions with NOI values ranging from 0.92 to 1 when tested against various phytopathogenic fungi [34]. In their study, in addition to the selected *S. cerevisiae* strains, several *T. delbrueckii* and *C. sake* strains also exhibited NOI values > 0.90, indicating a strong niche overlap. Although high NOI values were observed for *S. cerevisiae* and *T. delbrueckii* also in the present study, direct comparisons are not possible, as their analysis involved different fungal species (not limited to *B. cinerea*) and was based on a broader panel of 16 carbon sources, whereas the present study evaluated 14 carbon sources.

In contrast, Flores et al. (2024) assessed yeast niche overlap specifically against four *B. cinerea* strains using an even broader panel of carbon sources representative of table grape composition [35]. In that study, *S. cerevisiae* (BSc60) and *H. vineae* (BHv86) exhibited NOI values > 0.90 for all four tested *B. cinerea* strains, whereas other *S. cerevisiae* strains showed lower and more variable results. Notably, the two *T. delbrueckii* strains evaluated in their study exhibited only low NOI values [35].

### 3.9. Leaf Disc Assay

**Figure 9:**
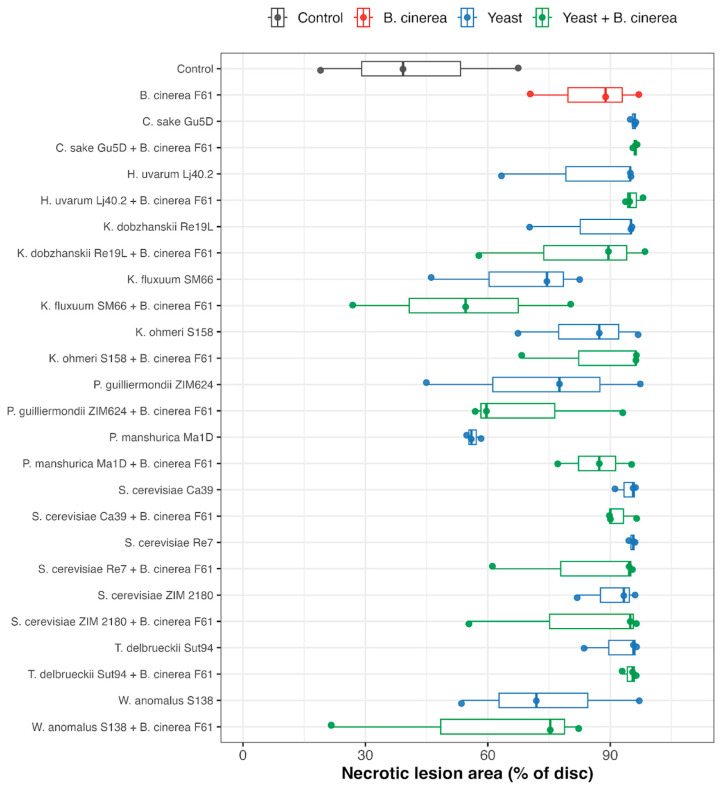
Necrotic lesion area on grapevine leaf discs after six days of incubation and DAB-staining. Boxplots show the medians and interquartile ranges with individual disc values overlaid. Colors indicate the treatment class (control, *B. cinerea* F61 or yeast alone, and combination yeast + *B. cinerea* F61).

Necrotic lesion severity in the leaf-disc assay was quantified by automated image analysis as the necrotic lesion area (% of disc), defined as the summed area of the two largest lesions per disc (Figure 9; Tables S8 and S9).

Substantial overlap in necrotic area distributions was observed among the control, *B. cinerea*, yeast alone, and yeast + *B. cinerea* treatments. The Kruskal–Wallis test indicated a moderate overall treatment effect (ε^2^ ≈ 0.18), but Dunn–Holm adjusted pairwise comparisons did not show statistically significant differences between treatments (all adjusted *p* ≥ 0.05; Table S9). This outcome is probably due to the limited number of replicates per treatment and the high inter-disc variability typical of late-stage lesion assessments. Nevertheless, several strains, like *K. ohmeri* S158, *P. guilliermondii* ZIM 624, *K. fluxuum* SM66, *W. anomalus* S138, and selected *S. cerevisiae* strains (Ca39, Re7) showed trends toward lower median necrotic lesion area or reduced variability compared with *B. cinerea* alone.

Overall, these data suggest that yeast treatments may modulate *B. cinerea*–associated lesion development, but the effects were not statistically separable at the six-day endpoint. Future experiments with increased replication and earlier or multi-timepoint sampling (with standardized imaging conditions and, if required, dedicated ROS-focused assays) will be important to resolve the timing and mechanism of any protective interactions.

### 3.10. Synthesis of Overall Functional Characteristics in the Best-Performing Strains

A stepwise, multi-criteria selection strategy resulted in a refined strain collection consisting of six non-*Saccharomyces* and two *Saccharomyces* strains, identified as a focused basis for further consideration and research. Importantly, for several of the selected strains, vinification relevance is supported by independent controlled fermentation experiments conducted in complementary studies by our research group [25,26,138].

***P. guilliermondii*** **ZIM 624** showed the strongest and most balanced overall profile among all evaluated isolates. This strain combined positive activity for all targeted enzymes with no detectable H_2_S production, high Y-MGRI values, strong VOC-mediated inhibition on both GJM and YPDA media, full fungicide tolerance, and a very high NOI (0.97), and was therefore selected for field evaluation. When tested in the vineyard in combination with complementary antifungal strategies, *P. guilliermondii* ZIM 624 significantly reduced disease incidence and severity in ‘Pinot noir’ compared to the controls, while ‘Pinot gris’ showed encouraging trends. Although these results indicate promising potential, they were obtained in a single, relatively dry vintage with low infection pressure, and further evaluation under higher disease risk conditions is required to confirm efficacy. Importantly, the relevance of *P. guilliermondii* ZIM 624 extends beyond the present screening and testing, as this strain was shown in an independent study conducted within our research group to significantly influence colour-related properties in Pinot noir skin extract and wine fermentations and to promote the formation of stable vinylphenolic pyranoanthocyanins, linking strain-specific metabolism to oenologically relevant outcomes [25].

In addition, ZIM 624 was independently demonstrated to produce volatile organic compounds with antifungal activity against *B. cinerea* in grape-based systems, directly supporting its biocontrol potential [36]. Moreover, this strain was evaluated in controlled sequential fermentation experiments using Sauvignon Blanc and Istrian Malvasia musts, as reported in conference communication [138]. It demonstrated a positive contribution to aroma development through enhanced varietal thiol release, thereby further substantiating its functional significance during vinification.

Several additional non-*Saccharomyces* strains displayed interesting complementary strengths.

***A. pullulans*** **ZIM2098** achieved the highest Y-MGRI values, together with relatively strong VOC-mediated inhibition, a broad enzymatic profile (except chitinase production), a high NOI, and good fungicide tolerance.

***T. delbrueckii*** **Sut94** exhibited a broad enzymatic profile (except chitinase) and very strong VOC-mediated inhibition coupled with a high NOI, although its lower Y-MGRI values and weak fungicide tolerance suggest a more context-dependent role.

***M. reukaufii*** **ZIM2019** displayed good enzymatic activity (except chitinolytic activity) and a high NOI, but only moderate Y-MGRI values and comparatively lower VOC-mediated inhibition, while remaining fungicide-tolerant [159]. This strain was previously shown to exhibit high anthocyanin adsorption capacity in Pinot noir skin extract fermentations, highlighting strong strain-dependent yeast–phenolic interactions [25].

***P. kluyveri*** **ZIM2031** showed a favourable enzymatic profile (except chitinase), high NOI, good Y-MGRI values (particularly on GJM), intermediate VOC-mediated inhibition, and good fungicide tolerance.

***P. manshurica*** **Ma1D** exhibited an interesting enzymatic profile (lacking only chitinase), moderate but stable Y-MGRI values on both media, high VOC-mediated inhibition on GJM, and good fungicide tolerance, supporting its inclusion among supplementary candidates.

***W. anomalus*** **S138**, initially identified as promising in an independent project conducted by our research group [159], was incorporated into segments of our study for validation. Its enzymatic performance was partially corroborated, and it demonstrated a unique enzymatic profile, including chitinase activity, which is uncommon among the tested strains. Consistent with previous findings, *W. anomalus* S138 was also shown to significantly influence pigment formation in Pinot noir skin extract fermentations, further supporting its prioritization [25].

***S. cerevisiae*** **Re7** and **ZIM2180** as representatives of *Saccharomyces strains*, ***S. cerevisiae*** **Re7** and **ZIM2180** showed a rather interesting enzymatic profile, strong VOC-mediated inhibition and high NOI values, together with generally good fungicide tolerance. However, growth performance was moderate, with Y-MGRI values declining over prolonged incubation, and trace H_2_S production was observed for ZIM2180. These strains are therefore considered more suitable as supporting or consortium components rather than primary biocontrol candidates.

Overall, favourable outcomes across NOI, VOC-mediated inhibition, Y-MGRI, fungicide tolerance, and leaf bioassays indicate—but do not confirm—the potential of the selected strains as biocontrol agents. Enzymatic profiling further suggests mechanistic functional potential, including chitinase activity (pathogen interaction), glycosidase activity (biocontrol and terpene release), *β*-lyase activity (thiol release), and sulfite reductase activity (risk of off-flavour formation).

When considered together with independent evidence from controlled Pinot noir extract and wine fermentations and complementary controlled fermentation experiments [25,26,138] this refined strain set provides a biologically justified and strategically focused foundation for subsequent validation under vineyard and vinification conditions.

### 3.11. A Field Trial Employing Selected Yeast in Combined Antifungal Strategies

Pinot grapevine varieties are among the most susceptible to *B. cinerea* infection because of their early ripening, which often coincides with harvest under warm and rainy conditions, and their compact cluster architecture [23,160].

**Figure 10:**
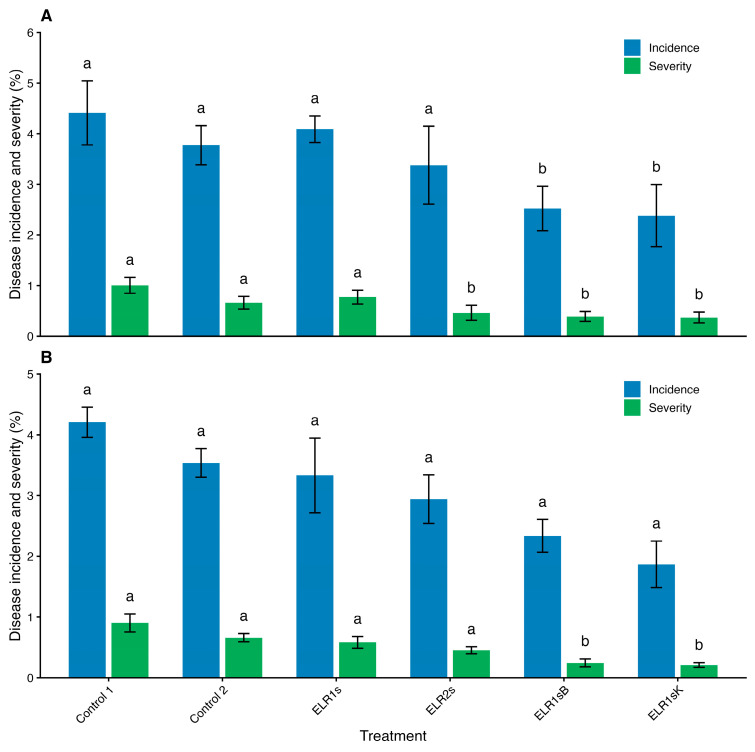
Incidence and severity (%) of *Botrytis cinerea* infection in ‘Pinot noir’ (**A**) and ‘Pinot gris’ (**B**) grapevine varieties. Values are expressed as mean ± SD (*n* = 3). Data were analysed by one-way ANOVA, and means were compared using Tukey’s HSD test (*p* < 0.05). Different letters indicate statistically significant differences among the treatments of each group. Legend: C1, control without leaf removal with one early application of Switch^®^; C2, control without leaf removal with two applications of Switch^®^; ELR1s, early leaf removal (ELR) with one early application of Switch^®^; ELR2s, ELR with two applications of Switch^®^; ELR1sB, ELR combined with a tested biocontrol agent and one early application of Switch^®^; ELR1sK, ELR combined with a commercial biocontrol agent and one early application of Switch^®^.

Figure 10 shows the results of a field trial conducted on two grapevine cultivars naturally prone to *B. cinerea* infection (‘Pinot gris’ and ‘Pinot noir’), comparing different combinations of antifungal strategies: canopy management, fungicide application, and biocontrol yeasts.

In Pinot cultivars, the complete avoidance of synthetic fungicide applications represents a risk that many winegrowers are unwilling to accept. Concurrently, concerns regarding fungicide residues in wine are prompting winemakers to restrict fungicide use to the early season only [23,161]. To address this limitation and support whole-season protection, this study focused on the potential of the safe omission of the second (late) fungicide application by integrating a combination of more sustainable disease management strategies.

In general, the results showed more promising outcomes for ‘Pinot noir’ than for ‘Pinot gris’. In the case of ‘Pinot noir’, both disease incidence (%) and severity (%) were significantly reduced in biocontrol-assisted treatments compared to the control treatments (Figure 10A).

Although pre-flowering (early) leaf removal (ELR) showed a positive trend in reducing disease severity, consistent with previous trials conducted in the same vineyard that resulted in an improved cluster architecture, microclimate or reduced *B. cinerea* infection [23,24], the effects observed in the present study did not reach statistical significance.

The exception, however only in ‘Pinot noir’, was ELR2s treatment, which showed comparable performance to the biocontrol-assisted treatments, suggesting that early leaf removal combined with two Switch^®^ applications could potentially be substituted with a single Switch^®^ application supported by a biocontrol agent. However, further experimental trials are required to substantiate this potential replacement strategy.

The effectiveness of pre-flowering leaf removal is attributed to reduced cluster compactness, which improves air circulation within the fruit zone and decreases the risk of berry damage caused by physical pressure between berries resulting from limited space during development [162,163]. Such structural cluster modifications in relation to early leaf removal have been previously documented in several grape varieties, including ‘Barbera’ and ‘Trebbiano’ [160], ‘Sauvignon blanc’ [164], ‘Pinot noir’ [23,165], ‘Pinot gris’ [162,166], and ‘Sangiovese’ [167,168].

Despite the absence of significant differences, the findings of the same study on ‘Pinot gris’ revealed encouraging trends in the incidence level in response to the implementation of yeast-mediated biocontrol. (Figure 10B). In contrast, when assessing infection severity, the findings revealed notable improvements in treatments incorporating both the experimental biocontrol yeast and the commercial variant yeast.

Although the results appear promising, it is crucial to acknowledge that the study was performed in only one vintage, which resulted in relatively dry conditions, minimizing the risk of infection. To thoroughly evaluate efficacy, further research is needed on vintages subjected to weather conditions that pose a higher risk of infection.

## 4. Conclusions

This study highlighted indigenous yeast isolates exhibiting functional traits with potential relevance to sustainable viticulture and winemaking. Among them, *P. guilliermondii* ZIM 624 exhibited the most balanced overall functional profile across the assessed parameters and was therefore selected for vineyard evaluation as a potential biocontrol agent against *B. cinerea*. Field experiments demonstrated significant reductions in *B. cinerea* incidence and severity in ‘Pinot noir’ compared to controls. In ‘Pinot gris’, incidence followed a similar yet non-significant trend, while a significant reduction in severity was observed. Further evaluation across multiple seasons and under more challenging climatic conditions is warranted to confirm the robustness and consistency of these effects. While the present work focused primarily on the vineyard performance of ZIM 624, its oenological relevance has been independently demonstrated in previous studies by our group, thus complementing the current findings. The combined evidence suggests the potential of ZIM 624 as a multifunctional candidate and supports the rationale for integrating vineyard biocontrol performance with fermentation-related functionality when selecting yeasts for practical application, particularly in winemaking contexts involving spontaneous fermentation. In addition, five non-*Saccharomyces* and two *Saccharomyces* strains exhibited specific functional strengths and, although individually less comprehensive than ZIM 624, may benefit from further evaluation under defined vineyard or winemaking conditions, including within mixed microbial strategies.

## Figures and Tables

**Table 1:** Qualitative determination of enzymatic activities for chitinase (Ch), glucoside hydrolase (GHA, GHC, GHE, GHS, GH 4-MUG), *β*-lyase (*β*L), and sulfite reductase (H_2_S) in experimental collection of yeast strains, using screening methods employing colloidal chitin; *β*-D-glucosidic substrates (arbutin (A), cellobiose (C), esculin (E), salicin (S), and 4-methylumbelliferyl-*β*-D-glucoside (4-MUG)); *S*-methyl-L-cysteine substrate; and lead acetate method in a microplate format.

Yeast Species	*n*	Ch	GHA	GHC	GHE	GHS	GH4-MUG	*β*L	H_2_S
*A. pullulans*	1	−	+	+	+	+	w	+	−
*B. albus*	1	+	+	+	+	+	−	+	−
*C. diversa*	1	−	+	+	+	+	−	−	−
*C. oleophila*	1	−	+	+	+	+	+	−	−
*C. vini*	1	−	+	+	+	+	+	−	−
*C. sake*	1	−	+	+	+	+	+	+	−
*C. zemplinina*	1	−	−	−	−	−	+	−	−
*D. hansenii*	1	−	w	+	+	w	+	+	−
*H. opuntiae*	1	−	+	w	+	+	+	+	−
*H. uvarum*	11	−	+	+	+	+	+	+(9/11)/n.d.	−(8/11)/w/n.d.
*K. servazzii* *(S. servazzii)*	2	−	−/w	−/w	−/w	−/w	+/−	+/−	−
*K. dobzhanskii*	1	−	+	+	+	+	+	+	−
*K. fluxuum*	1	−	+	+	+	+	+	+	−
*K. ohmeri*	1	−	+	+	+	w	+	n.d.	n.d.
*L. thermotolerans*	8	−	+(6/8)/v	+(6/8)/w	+(7/8)/w	v	+(7/8)/−	+(6/8)/n.d.	−(5/8)/w
*M. fructicola*	1	−	+	+	+	+	+	+	−
*M. pulcherrima*	2	−/w	+/w	+/w	+	+/w	+/w	w/n.d.	−/n.d.
*M. reukaufii*	1	−	+	+	+	+	+	+	−
*P. guilliermondii*	2	+	+	+	+	+	+	+	−
*P. kluyveri*	3	−	+	+(2/3)/−	+	+(2/3)/−	v	+(2/3)/n.d.	−(2/3)/n.d.
*P. kudriavzevii*	3	−(2/3)/w	+(2/3)/w	+	w(2/3)/+	+	+	v	−(2/3)/n.d.
*P. manshurica*	14	−	+(13/14)/−	+(13/14)/−	+(13/14)/−	+(13/14)/−	+(13/14)/−	+(13/14)/−	v
*P. membranifaciens*	1	−	+	+	+	+	+	+	−
*H. valbyensis*	1	−	+	+	+	+	n.d.	n.d.	n.d.
*S. bayanus*	1	−	−	−	+	−	+	−	w
*S. cerevisiae*	17	−	+(16/17)/w	+(16/17)/w	+(15/17)/w	+(15/17)/w	+(16/17)/w	+(16/17)/w	−(16/17)/w
*S. kudriavzevii*	7	−	+	+	+	+	+	+	−
*S. paradoxus*	21	−	+(20/21)/w	+(20/21)/w	+(20/21)/w	+(20/21)/w	+(20/21)/w	+(20/21)/−	−
*T. delbrueckii*	7	−	+(6/7)/−	+(6/7)/w	+(6/7)/w	+(6/7)/w	+	+	−
*W. anomalus*	1	+	+	+	+	+	+	n.d.	n.d.

Legend: *n* (number of strains); + (positive); − (negative); w (weak); n.d. (not determined); v (variable results (+, −, and w; or in cases where only two outcomes were obtained, the numbers of strains are presented accordingly)). The experimental findings are derived from a minimum of three independent replicates.

## Data Availability

The original contributions presented in the study are included in the article/Supplementary Materials; further inquiries can be directed to the corresponding authors.

## Supplementary Materials

The following supporting information can be downloaded at https://www.mdpi.com/article/10.3390/microorganisms14030705/s1; Table S1: List of yeast isolates used in the study; Table S2: Qualitative determination of encymatic activities of individual strains; Table S3: Pairwise non-parametric comparisons of radial growth inhibition (%) between yeast isolates cultivated on GJM and YPDA media at the indicated observation time points (7 and 30 days); Table S4: Comparison of yeast-mediated radial growth inhibition of Botrytis cinerea between day 7 and day 30 of incubation; Table S5: Pairwise comparisons of inhibitory activity of yeast-modulated antifungal volatile organic compounds (VOCs); Table S6: Comparison of yeast-mediated antifungal activity of volatile organic compounds (VOCs) between grape juice medium (GJM) and yeast extract–peptone–dextrose agar (YPDA); Table S7: Yeasts tolerance to different CuSO4 concentrations, iprodione (Rovral^TM^ Aquaflo), cyprodinil/fludioxonil combination (Switch 62.5WG), Rovral and Banjo on solid media; Table S8: Summary statistics of necrotic lesion area (% of disc) by treatment in the grapevine leaf-disc assay, quantified by automated image analysis as the sum of the two largest lesions per disc; Table S9: Pairwise comparisons of necrotic lesion area (% of disc) between treatments using Dunn–Holm adjusted *p*-values following a Kruskal–Wallis test; Figure S1: Taxonomic distribution of enzymatic traits across yeast families; Figure S2: Family-level distribution of Yeast-mediated Fungal Mycelial Radial Growth Inhibition (Y-FMGRI, %) on GJM and YPDA media; Figure S3: Family-level distribution of VOC-mediated inhibition (%) against *B. cinerea* on GJM and YPDA media.
