# Supplementary material for: Harnessing the Enzymatic Potential of Indigenous Yeast Strains: Screening and Evaluation for Biocontrol and Oenological Advancements
Source: Microorganisms. 2026 Mar 21;14(3):705. doi: 10.3390/microorganisms14030705 (PMC13029107; doi:10.3390/microorganisms14030705)
Supplement: Supplementary file 1 [file microorganisms-14-00705-s001.zip › Supplementary_material_Figure_S1.pdf]

microorganisms

MDPI

Supplementary Material

Harnessing the enzymatic potential of indigenous yeast strains: screening and evaluation for biocontrol and oenological advancements

Rowland Adetayo Adesida, Jan Reščič, Lorena Butinar* and Melita Sternad Lemut*

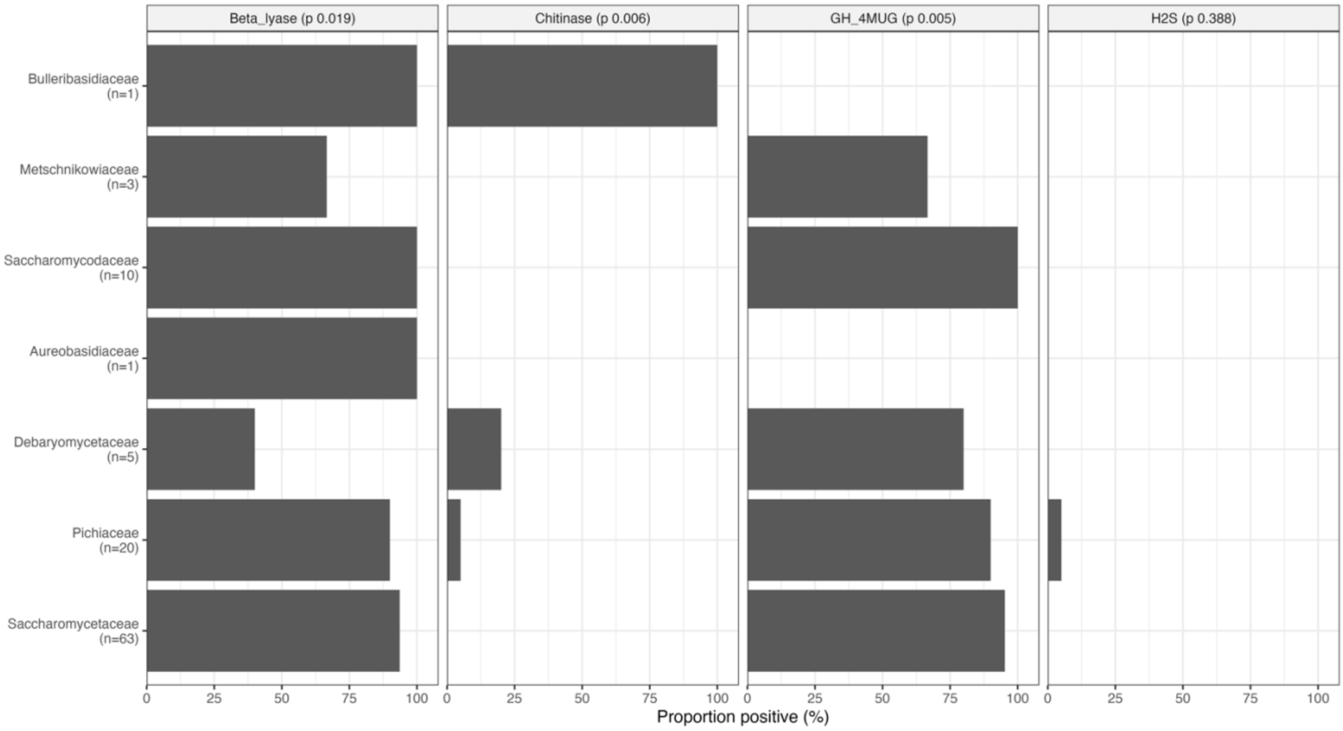

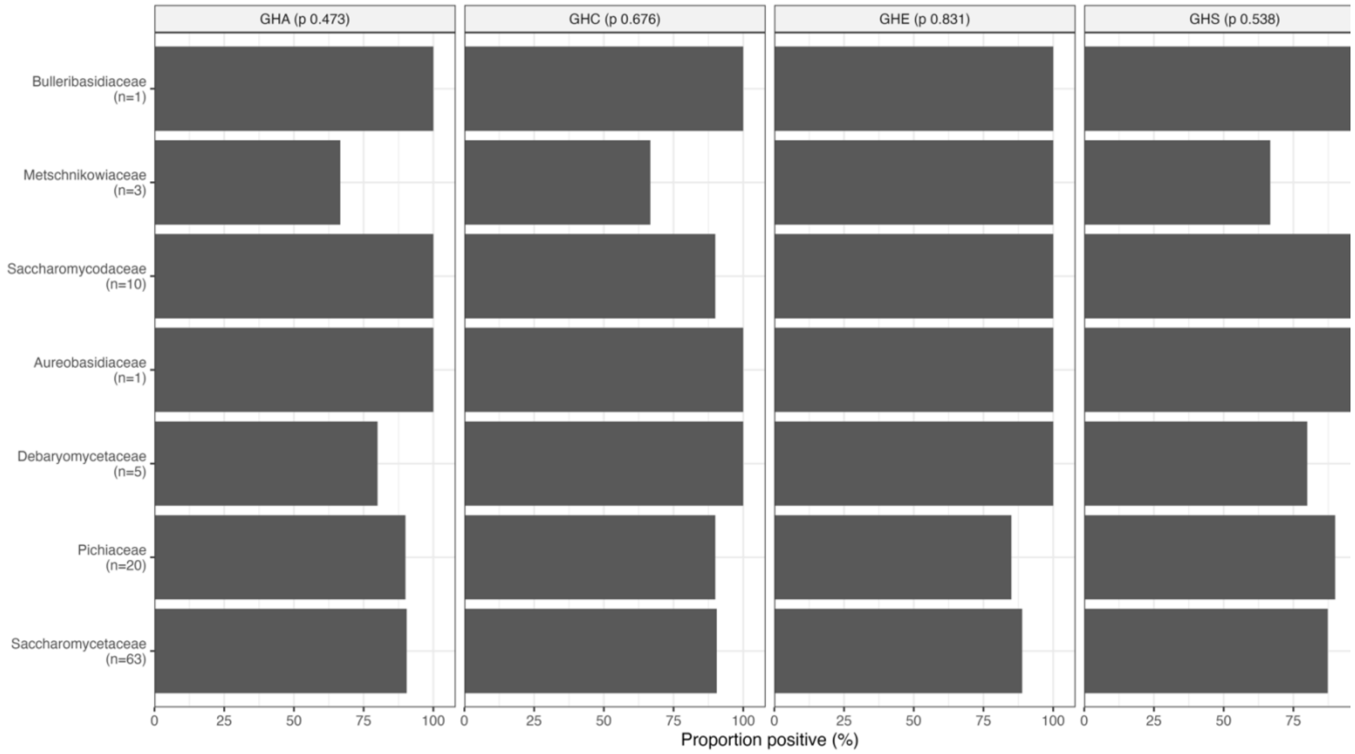

Figure S1. Taxonomic distribution of enzymatic traits across yeast families. Proportion of positive strains (%) for glycosidase activities (GHA, GHC, GHE, GHS; upper panel) and additional enzymatic traits (β-lyase, chitinase, GH_4MUG, and H_2S production; lower panel) across yeast families. Bars represent the percentage of positive strains within each family; sample size per family is indicated in parentheses. P-values shown in the strip headers correspond to Kruskal–Wallis tests assessing differences among families for each enzymatic trait.
