# Supplementary material for: Harnessing the Enzymatic Potential of Indigenous Yeast Strains: Screening and Evaluation for Biocontrol and Oenological Advancements
Source: Microorganisms. 2026 Mar 21;14(3):705. doi: 10.3390/microorganisms14030705 (PMC13029107; doi:10.3390/microorganisms14030705)
Supplement: Supplementary file 1 [file microorganisms-14-00705-s001.zip › Supplementary_material_Figure_S3.pdf]

microorganisms

Supplementary Material

Harnessing the enzymatic potential of indigenous yeast strains: screening and evaluation for biocontrol and oenological advancements

Rowland Adetayo Adesida, Jan Reščič, Lorena Butinar* and Melita Sternad Lemut*

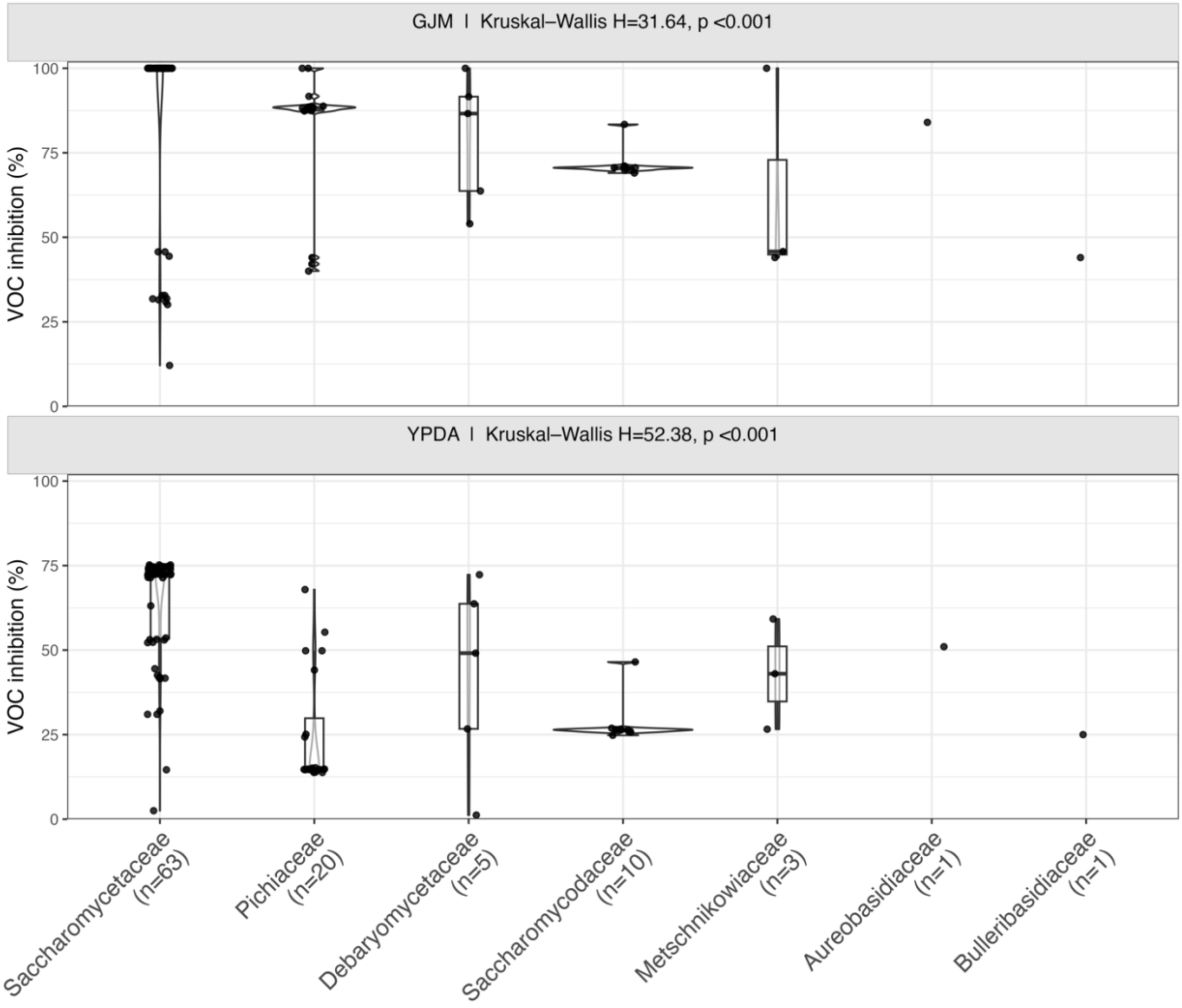

Figure S3. Family-level distribution of VOC-mediated inhibition (%) against *B. cinerea* on GJM and YPDA media. Violin plots depict the distribution of percentage inhibition across yeast families, with boxplots indicating median and interquartile range and individual strains shown as points. Only families with ≥2 strains are represented by violin and boxplots; families with n = 1 are shown as single data points. Inhibition values are expressed as percentages (0–100%). Kruskal–Wallis statistics are displayed in the grey header for each media.
