# Supplementary material for: Harnessing the Enzymatic Potential of Indigenous Yeast Strains: Screening and Evaluation for Biocontrol and Oenological Advancements
Source: Microorganisms. 2026 Mar 21;14(3):705. doi: 10.3390/microorganisms14030705 (PMC13029107; doi:10.3390/microorganisms14030705)
Supplement: Supplementary file 1 [file microorganisms-14-00705-s001.zip › Supplementary_material_Table_S1.pdf]

Table S1. List of yeast isolates used in the study

Yeast isolate strain designation	Genus	Species	Origin	Locality
Ca1	*Saccharomyces*	*cerevisiae*	Vineyard; vine bark	Neblo, Brda, Slovenia
Ca11	*Saccharomyces*	*paradoxus*	Vineyard; oak bark	Neblo, Brda, Slovenia
Ca15	*Saccharomyces*	*cerevisiae*	Vineyard; oak bark	Neblo, Brda, Slovenia
Ca22L	*Pichia*	*manshurica*	Vineyard; soil	Neblo, Brda, Slovenia
Ca29L	*Pichia*	*manshurica*	Vineyard; oak bark and leaf	Neblo, Brda, Slovenia
Ca39	*Saccharomyces*	*cerevisiae*	Vineyard; soil under oak tree	Neblo, Brda, Slovenia
Ca81	*Meyerozyma (Pichia)*	*guilliermondii*	Vineyard; leaf, bark, twig, berry from vine plant	Neblo, Brda, Slovenia
Ca95	*Saccharomyces*	*servazzii*	Vineyard; grapes	Neblo, Brda, Slovenia
Ca109	*Saccharomyces*	*paradoxus*	Vineyard; oak bark	Neblo, Brda, Slovenia
Ca127	*Kazachstania*	*servazzii*	Vineyard; soil under oak tree	Neblo, Brda, Slovenia
Ca144	*Saccharomyces*	*cerevisiae*	Vineyard; grapes and insects	Neblo, Brda, Slovenia
Ca146	*Saccharomyces*	*cerevisiae*	Vineyard; grapes	Neblo, Brda, Slovenia
Ca147	*Hanseniaspora*	*uvarum*	Vineyard; grapes	Neblo, Brda, Slovenia
F29	*Saccharomyces*	*paradoxus*	Forest; soil under oak tree	Nanos slopes, Slovenia
F45	*Saccharomyces*	*kudriavzevii*	Forest; soil under oak tree	Nanos slopes, Slovenia
F48	*Saccharomyces*	*servazzii*	Forest; oak bark	Nanos slopes, Slovenia

F60	*Saccharomyces*	*paradoxus*	Forest; soil under oak tree	Nanos slopes, Slovenia
F67	*Saccharomyces*	*paradoxus*	Forest; oak bark	Nanos slopes, Slovenia
Gu36	*Saccharomyces*	*cerevisiae*	3 days old fermenting must from the cellar	Planina, Slovenia
Gu5D	*Candida*	*sake*	Vineyard; soil	Planina, Slovenia
Gu50	*Saccharomyces*	*cerevisiae*	Vineyard; oak bark	Planina, Slovenia
Gu60	*Saccharomyces*	*cerevisiae*	Leftovers of white grape skins after pressing	Planina, Slovenia
Gu81	*Lachancea*	*thermotolerans*	Vineyard; grapes	Planina, Slovenia
Gu94	*Saccharomyces*	*kudriavzevii*	Vineyard; soil under oak tree	Planina, Slovenia
Gu95	*Saccharomyces*	*paradoxus*	Vineyard; leaves and twig of the oak tree	Planina, Slovenia
Lj40.2	*Hanseniaspora*	*uvarum*	Vineyard; grapes	Lijak, Slovenia
Lj52	*Hanseniaspora*	*uvarum*	Forest; Oak	Lijak, Slovenia
M11D	*Pichia*	*manshurica*	Vineyard; soil	Dobravlje, Slovenia
M22	*Saccharomyces*	*paradoxus*	Vineyard; soil	Dobravlje, Slovenia
M49	*Pichia*	*manshurica*	Vineyard; leaves, bark, stem of the oak tree	Dobravlje, Slovenia
M88	*Saccharomyces*	*cerevisiae*	Vineyard; grape cluster stems	Dobravlje, Slovenia
M90	*Saccharomyces*	*cerevisiae*	Vineyard; soil under oak tree	Dobravlje, Slovenia
M94	*Saccharomyces*	*kudriavzevii*	Vineyard; soil under oak tree	Dobravlje, Slovenia
Ma1D	*Pichia*	*manshurica*	Vineyard; vine bark	Manče, Slovenia
Ma1L	*Torulaspora*	*delbrueckii*	Vineyard; vine bark	Manče, Slovenia
Ma4L	*Pichia*	*manshurica*	Vineyard; soil	Manče, Slovenia

Ma11D	*Pichia*	*manshurica*	Vineyard; oak	Manče, Slovenia
Ma11L	*Pichia*	*manshurica*	Vineyard; oak	Manče, Slovenia
Ma35	*Hanseniaspora*	*uvarum*	Vineyard; grapes	Manče, Slovenia
Ma40	*Pichia*	*kudriavzevii*	Vineyard; soil	Manče, Slovenia
Ma41	*Hanseniaspora*	*uvarum*	Vineyard; grapes	Manče, Slovenia
Ma42	*Hanseniaspora*	*uvarum*	Vineyard; soil under oak tree	Manče, Slovenia
Ma59	*Torulaspora*	*delbrueckii*	Vineyard; soil	Manče, Slovenia
Ma101	*Saccharomyces*	*kudriavzevii*	Vineyard; oak bark	Manče, Slovenia
Ma106	*Saccharomyces*	*paradoxus*	Vineyard; oak bark	Manče, Slovenia
Ma108	*Saccharomyces*	*paradoxus*	Vineyard; soil under oak tree	Manče, Slovenia
M3D	*Pichia*	*manshurica*		
Re6	*Torulaspora*	*delbrueckii*	Vineyard; vine bark	Dutovlje (Kras), Slovenia
Re7	*Saccharomyces*	*cerevisiae*	Vineyard; cherry fruit	Dutovlje (Kras), Slovenia
Re18_D	*Lachancea*	*thermotolerans*	Vineyard; oak bark	Dutovlje (Kras), Slovenia
Re19L	*Kluyveromyces*	*dobzhanskii*	Vineyard; soil under oak tree	Dutovlje (Kras), Slovenia
SM10	*Saccharomyces*	*paradoxus*	Vineyard; soil	Brje, Slovenia
SM10L	*Pichia*	*manshurica*	Vineyard; soil	Brje, Slovenia
SM11	*Saccharomyces*	*paradoxus*	Vineyard; vine bark	Brje, Slovenia
SM26L	*Pichia*	*manshurica*	Vineyard; vine leaf	Brje, Slovenia
SM42L	*Saccharomyces*	*paradoxus*	Wine	Brje, Slovenia

SM36	*Torulaspora*	*delbrueckii*	Vineyard; vine bark and leaf	Brje, Slovenia
SM58	*Saccharomyces*	*paradoxus*	Soil under oak tree	Brje, Slovenia
SM66	*Kregervanrija*	*fluxuum*	Soil under oak tree	Brje, Slovenia
SM73	*Hanseniaspora*	*uvarum*	Vineyard; vine bark and leaves	Brje, Slovenia
SM76.2	*Saccharomyces*	*cerevisiae*	Vineyard; grapes and insects	Brje, Slovenia
SM113	*Saccharomyces*	*paradoxus*	Soil under oak tree	Brje, Slovenia
Sut42	*Hanseniaspora*	*opuntiae*	Vineyard; vine bark, leaf and twig	Podraga, Slovenia
Sut21	*Torulaspora*	*delbrueckii*	Wine	Podraga, Slovenia
Sut45	*Pichia*	*kluyveri*	Vineyard; vine bark, leaf and twig	Podraga, Slovenia
Sut75	*Saccharomyces*	*cerevisiae*	Oak leaves and twig, near the vineyard	Podraga, Slovenia
Sut80	*Hanseniaspora*	*uvarum*	Oak leaves and twig, near the vineyard	Podraga, Slovenia
Sut82	*Saccharomyces*	*cerevisiae*	Vineyard; oak bark	Podraga, Slovenia
Sut85	*Saccharomyces*	*paradoxus*	Vineyard; soil under the oak tree	Podraga, Slovenia
Sut86	*Saccharomyces*	*paradoxus*	Vineyard; oak bark	Podraga, Slovenia
Sut91.2	*Pichia*	*manshurica*	Vineyard; soil under the oak tree	Podraga, Slovenia
Sut94	*Torulaspora*	*delbrueckii*	Vineyard; vine bark	Podraga, Slovenia
Sut99	*Saccharomyces*	*paradoxus*	Vineyard; soil under the oak tree	Podraga, Slovenia
Sut103	*Saccharomyces*	*paradoxus*	Vineyard; oak bark	Podraga, Slovenia
Sut104	*Metschnikowia*	*fructicola*	Vineyard; oak bark, leaf and twig	Podraga, Slovenia

Sut106	*Saccharomyces*	*kudriavzevii*	Vineyard; soil under the oak tree	Podraga, Slovenia
Sut116	*Debaryomyces*	*hansenii*	Grape pomace from the cellar	Podraga, Slovenia
Sut127	*Pichia*	*kudriavzevii*	Wine	Podraga, Slovenia
SV21	*Saccharomyces*	*cerevisiae*	Oak bark	Sinji vrh, Slovenia
SV22	*Torulaspora*	*delbrueckii*	Oak bark	Sinji vrh, Slovenia
SV31	*Lachancea*	*thermotolerans*	Soil under the oak tree	Sinji vrh, Slovenia
SV33	*Lachancea*	*thermotolerans*	Oak bark	Sinji vrh, Slovenia
SV34	*Lachancea*	*thermotolerans*	Soil under the oak tree	Sinji vrh, Slovenia
SV37D	*Pichia*	*manshurica*	Soil under the oak tree	Sinji vrh, Slovenia
SV37L	*Pichia*	*manshurica*	Soil under the oak tree	Sinji vrh, Slovenia
SV96	*Saccharomyces*	*paradoxus*	Oak bark	Sinji vrh, Slovenia
V5	*Saccharomyces*	*paradoxus*	Oak bark	Vipava, Slovenia
V6	*Lachancea*	*thermotolerans*	Soil under the oak tree	Vipava, Slovenia
V34	*Saccharomyces*	*cerevisiae*	Oak bark	Vipava, Slovenia
V64	*Saccharomyces*	*kudriavzevii*	Soil under the oak tree	Vipava, Slovenia
V71	*Saccharomyces*	*paradoxus*	Soil under the oak tree	Vipava, Slovenia
ZIM 0608	*Bulleromyces*	*albus*	Grape berries of Rebula without fungicide application	Goriška brda, Slovenia
ZIM 0624	*Pichia*	*guilliermondii*	Grape berries of Rebula treated with pyrimethanil	Goriška brda, Slovenia

ZIM 0670	*Hanseniaspora*	*uvarum*	Must of Rebula – spontaneous fermentation; grape without fungicide application	Goriška brda, Slovenia
ZIM 0674	*Candida*	*zemplinina*	Must of Rebula – spontaneous fermentation	Goriška brda, Slovenia
ZIM 0707	*Pichia*	*membranifaciens*	Must of Rebula – spontaneous fermentation	Goriška brda, Slovenia
ZIM 2019	*Metschnikowia*	*reukaufii*	Surface of Modra frankinja grape berry	Pleterje, Slovenia
ZIM 2031	*Pichia*	*kluyveri*	Surface of Modra frankinja grape berry	Nemška gora, Slovenia
ZIM 2055	*Metschnikowia*	*pulcherrima*	Surface of Modra frankinja grape berry	Hom, Slovenia
ZIM 2098	*Aureobasidium*	*pullulans*	Surface of Žametna črnina grape berry	Trška gora, Slovenia
ZIM 2110	*Candida*	*diversa*	Must of Kraljevina	Agricultural Institute of Slovenia, Slovenia
ZIM 2122	*Saccharomyces*	*bayanus*	Must of Žametna črnina	Ljubljana, Slovenia
ZIM 2180	*Saccharomyces*	*cerevisiae*	Must of Kraljevina – spontaneous fermentation	Agricultural Institute of Slovenia, Slovenia
ZIM 2276	*Candida*	*oleophila*	Fruit of Olea europea (olive)	
ZIM 2305	*Candida*	*vini*	Spoiled Wine (Cviček)	Slovenia
116	*Hanseniaspora*	*uvarum*	Vineyard, vine, grape; integrated pest management	Manzano, Italy
115	*Lachancea*	*thermotolerans*	Vineyard, vine, grape; integrated pest management	Manzano, Italy
126	*Candida/* *Starmerella* spp.		Vineyard, vine, grape; organic pest management	Gramogliano, Italy
129	*Candida*	*tropicalis*	Vineyard, vine, grape; organic pest management	Cero, Italy

130	*Candida*	*tropicalis*	Vineyard, vine, grape; organic pest management	Cero, Italy
131	*Pichia*	*terricola*	Vineyard, vine, grape; advanced integrated pest management	Novacuzzo, Italy
132	*Lachancea*	*thermotolerans*	Vineyard, vine, grape; advanced integrated pest management	Novacuzzo, Italy
138	*Wickerhamomyces*	*anomalus*	Vineyard, vine, grape; advanced integrated pest management	Zuccole, Italy
139	*Metschnikowia*	*pulcherrima*	Vineyard, vine, grape; advanced integrated pest management	Zuccole, Italy
158	*Kodamaea*	*ohmeri*	Vineyard, soil, organic pest management	Gramogliano, Italy
163	*Pichia*	*kudriavzevii*	Vineyard, vine, grape; advanced integrated pest management	Novacuzzo, Italy
IT_VR54	*Pichia*	*kluyveri*	Vineyard, soil; integrated pest management	Capriva, Italy
IT_Ve78	*Hanseniaspora*	*valbyensis*	Grape must; organic pest management	Cero, Italy
IT_Ve77	*Hanseniaspora*	*uvarum*	Grape must; organic pest management	Cero, Italy
