# Supplementary material for: Harnessing the Enzymatic Potential of Indigenous Yeast Strains: Screening and Evaluation for Biocontrol and Oenological Advancements
Source: Microorganisms. 2026 Mar 21;14(3):705. doi: 10.3390/microorganisms14030705 (PMC13029107; doi:10.3390/microorganisms14030705)
Supplement: Supplementary file 1 [file microorganisms-14-00705-s001.zip › Supplementary_material_Table_S2.pdf]

microorganisms

Supplementary Material

Harnessing the enzymatic potential of indigenous yeast strains: screening and evaluation for biocontrol and oenological advancements

Rowland Adetayo Adesida, Jan Reščič, Lorena Butinar* and Melita Sternad Lemut*

Table S2. Qualitative determination of encimatic activities for *a)* chitinase (**Ch**), *b)* glucoside hydrolase (GH**E, GHA, GHS, GHC, GH4-MUG**), *c) β*-lyase (*β*L) and *d)* sulfite reductase (H_2S) in experimental collection of indegiuonous yeast strains using screening methods based on: *a)* chitin; *b)* solid medium supplemented with *β*-D-glucosides (esculin, arbutin, salicin, cellobiose, 4-methylumbelliferyl-*β*-D-glucoside (4-MUG)); *c) S*-methyl-L-cysteine; *d)* lead acetate method in a microplate format.

Yeast species	No. of strains	Strain designation	Ch	GHE	GHA	GHS	GHC	GH 4-MUG	βL	H_2S
A. pullulans	1	ZIM2098	-	+	+	+	+	w	+	-
B. albus	1	ZIM0608	+	+	+	+	+	-	+	-
C. diversa	1	ZIM2110	-	+	+	+	+	-	-	-
C. oleophila	1	ZIM2276	-	+	+	+	+	+	-	-
C. vini (K. fluxuum)	1	ZIM2305	-	+	+	+	+	+	-	-
C. sake	1	Gu 5D	-	+	+	+	+	+	+	-
C. zemplinina	1	ZIM0674	-	-	-	-	-	+	-	-
D. hansenii	1	Sut116	-	+	w	w	+	+	+	-
H. opuntiae	1	Sut42	-	+	+	+	w	+	+	-
H. uvarum	11	ZIM0670	-	+	+	+	+	+	+	w
		Lj40.2	-	+	+	+	+	+	+	-
		Lj52, Ma35, Ma41, Ma42, SM73, Sut80, Ca147	-	+	+	+	+	+	+	-
		116, IT_Ve77	-	+	+	+	+	+	n.d.	n.d.
K. servazzii	2	Ca95	-	w	w	w	w	+	-	-
		F48	-	-	-	-	-	-	+	-
K. dobzhanskii	1	Re19L	-	+	+	+	+	+	+	-
K. fluxuum	1	SM66	-	+	+	+	+	+	+	-
K. ohmeri	1	158	-	+	+	w	+	+	n.d.	n.d.
L. thermotolerans	8	Gu81, SV31, SV33, V6	-	+	+	+	+	+	+	-
		Re18_D	-	+	+	+	+	+	+	w
		SV34	-	+	+	w	+	+	+	-
		115	-	w	-	w	w	-	n.d.	n.d.
		132	-	+	w	-	w	+	n.d.	n.d.
M. fructicola	1	Sut104	-	+	+	+	+	+	+	-
M. pulcherrima	2	ZIM2055	w	+	w	w	w	w	w	-
		139	-	+	+	+	+	+	n.d.	n.d.
M. reukaufii	1	ZIM2019	-	+	+	+	+	+	+	-
P. guilliermondii	2	ZIM0624, Ca81	+	+	+	+	+	+	+	-
P. kluyveri	3	ZIM2031	-	+	+	+	+	+	+	-
		Sut45.1	-	+	+	-	-	-	+	-
		IT_VR54	-	+	+	+	+	n.d.	n.d.	n.d.
P. kudriavzevii	3	Ma40	w	w	w	+	+	+	-	-
		Sut127	-	w	+	+	+	+	+	-
		163	-	+	+	+	+	+	n.d.	n.d.

P. manshurica	14	Ca22L	-	-	-	-	-	-	-	-
		Ca29L, M11D, Ma1D, Ma4L, Ma11D, Ma11L, M3D, SM10L, SM26L, SV37D, SV37L	-	+	+	+	+	+	+	-
		M49	-	+	+	+	+	+	+	w
		Sut91.2	-	+	+	+	+	+	+	+
P. membranifaciens	1	ZIM0707	-	+	+	+	+	+	+	-
H. valbyensis	1	IT_Ve78	-	+	+	+	+	n.d.	n.d.	n.d.
S. bayanus	1	ZIM2122	-	+	-	-	-	+	-	w
S. cerevisiae	17	Ca1	-	w	w	w	w	w	w	-
		Ca15, Ca39, Ca144, Ca146, Gu36, Gu50, Gu60, M88, M90, Re7, SM76, Sut75, Sut82, SV21, V34	-	+	+	+	+	+	+	-
		ZIM2180	-	w	+	w	+	+	+	w
S. kudriavzevii	7	Ca127, F45, Gu94, M94, Ma101, Sut106, V64	-	+	+	+	+	+	+	-
S. paradoxus	21	Ca109	-	+	+	+	+	+	-	-
		Ca11, F60, F67, Gu95, M22, MA106, SM10, SM11, SM113, SM58, Sut103, Sut85, Sut86, Sut99, SV96, V5, V71, SM42L	-	+	+	+	+	+	+	-
		MA108	-	+	+	+	+	w	+	-
		F29	-	w	w	w	w	+	+	-
T. delbrueckii	7	Ma1L	-	w	-	w	w	+	+	-
		Ma59, Re6, SM36, Sut21, Sut94, SV22	-	+	+	+	+	+	+	-
W. anomalus	1	138	+	+	+	+	+	+	n.d.	n.d.

Legend: + (positive); - (negative); w (weak); n.d. (not determined). The experimental findings represent the results of at least three independent replicates.
