# Supplementary material for: Harnessing the Enzymatic Potential of Indigenous Yeast Strains: Screening and Evaluation for Biocontrol and Oenological Advancements
Source: Microorganisms. 2026 Mar 21;14(3):705. doi: 10.3390/microorganisms14030705 (PMC13029107; doi:10.3390/microorganisms14030705)
Supplement: Supplementary file 1 [file Supplementary_material_Figure_S2.pdf › Supplementary_material_Figure_S2.pdf]

microorganisms

MDPI

*Supplementary Material*

# Harnessing the enzymatic potential of indigenous yeast strains: screening and evaluation for biocontrol and oenological advancements

**Rowland Adetayo Adesida, Jan Reščič, Lorena Butinar* and Melita Sternad Lemut***

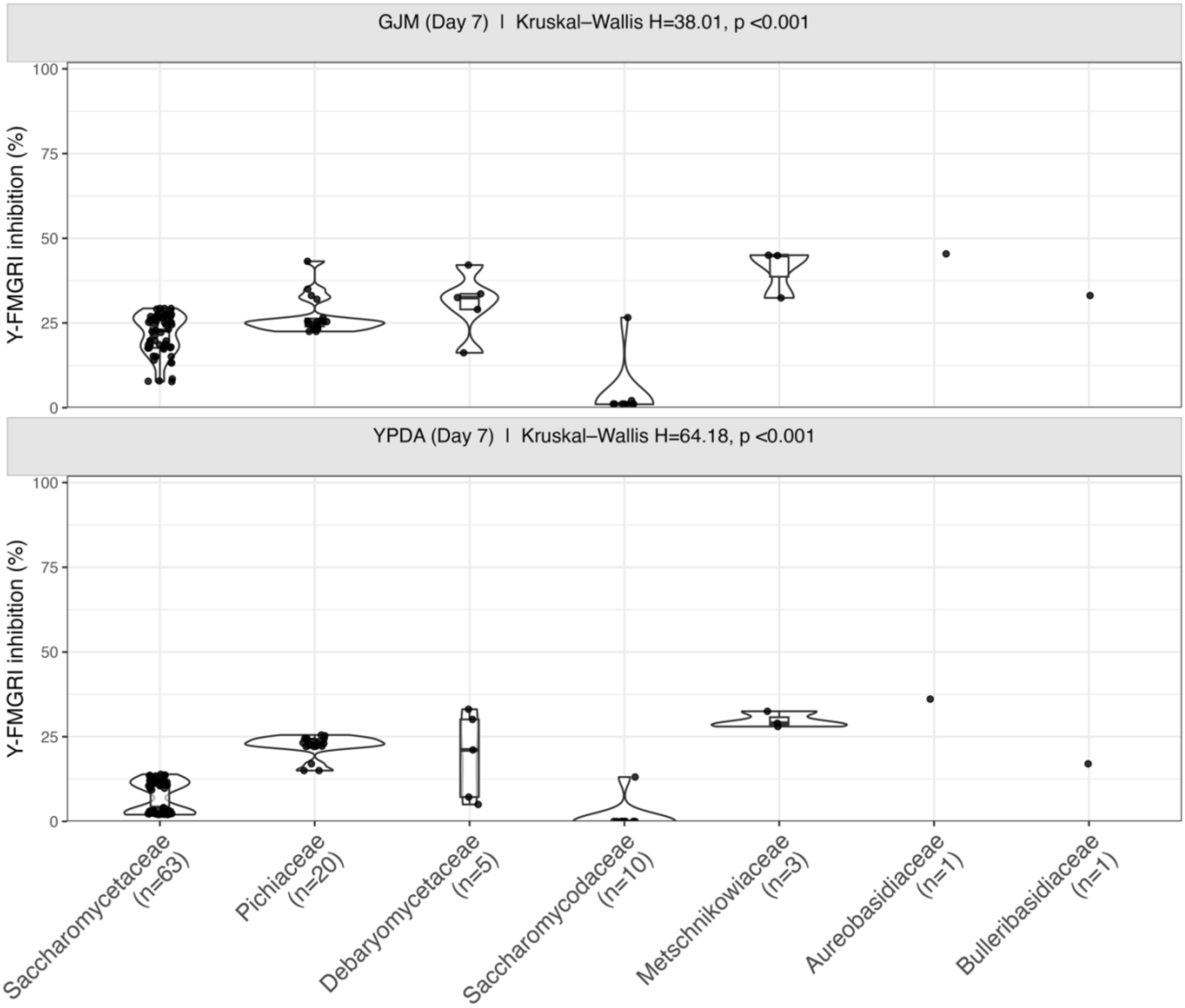


**Figure S2. Family-level distribution of Yeast-mediated Fungal Mycelial Radial Growth Inhibition (Y-FMGRI, %) on GJM and YPDA media (on day 7).** Violin plots show the distribution of inhibition percentages across yeast families, with boxplots indicating median and interquartile range; individual strains are overlaid as points. Only families with ≥2 strains are represented by violin/boxplots; families with n=1 are shown as single data points. Kruskal–Wallis statistics are displayed in the grey header for each medium.
